# Chewing lice of wild birds in Iran: new data and a checklist of avian louse species reported in Iran

**DOI:** 10.3389/fvets.2023.1324619

**Published:** 2024-02-02

**Authors:** Zahra Bahiraei, Alireza Sazmand, Javad Khedri, Mohammad Babaei, Erfan Moeinifard, Bilal Dik

**Affiliations:** ^1^Department of Pathobiology, Faculty of Veterinary Medicine, Bu-Ali Sina University, Hamedan, Iran; ^2^Department of Pathobiology, Faculty of Veterinary Medicine, Ferdowsi University, Mashhad, Iran; ^3^Department of Clinical Sciences, Faculty of Veterinary Medicine, Bu-Ali Sina University, Hamedan, Iran; ^4^Provincial Department of Environment, Hamedan, Iran; ^5^Department of Parasitology, Veterinary Faculty, Selçuk University, Konya, Türkiye

**Keywords:** birds of prey, chewing louse species, fauna, host–parasite associations, Iran, Middle-east, new record, Phthiraptera

## Abstract

Between September 2019 and December 2023, a total of 612 wild birds representing 16 orders, 33 families, 60 genera, and 78 species from nine provinces of Iran with different climates namely Hamedan (*n* = 54), Sistan-va-Baluchestan (*n* = 372), Kerman (*n* = 73), South Khorasan (*n* = 52), Mazandaran (*n* = 7), Chaharmahal-va-Bakhtiari (*n* = 2), Gilan (*n* = 2), Golestan (*n* = 18), North Khorasan (*n* = 9), and Razavi Khorasan (*n* = 23) were examined for chewing lice infestation. Naked eye examination revealed that 58 birds (9.5%) were infested with at least one chewing louse species. Collected lice specimens belonged to 28 species from the families Philopteridae, Menoponidae and Laemobothriidae including *Strigiphilus strigis* (*n* = 55, 15.6%), *Falcolipeurus quadripustulatus* (*n* = 41, 11.6%), *Craspedorrhynchus platystomus* (*n* = 40, 11.3%), *Colpocephalum turbinatum* (*n* = 36, 10.2%), *Laemobothrion maximum* (*n* = 25, 7.1%), *Nosopon lucidum* (*n* = 20, 5.6%), *Degeeriella fulva* (*n* = 18, 5.1%), *Colpocephalum eucarenum* (*n* = 16, 4.5%), *Laemobothrion vulturis* (*n* = 15, 4.2%), *Anaticola crassicornis* (*n* = 13, 3.7%), *Craspedorrhynchus aquilinus* (*n* = 9, 2.5%), *Degeeriella fusca* (*n* = 7, 2.0%), *Aegypoecus trigonoceps* (*n* = 7, 2.0%), *Quadraceps obscurus* (*n* = 6, 1.7%), *Colpocephalum impressum* (*n* = 6, 1.7%), *Trinoton querquedulae* (*n* = 6, 1.7%), *Colpocephalum heterosoma* (*n* = 5, 1.4%), *Colpocephalum nanum* (*n* = 5, 1.4%), *Lunaceps holophaeus* (*n* = 4, 1.1%), *Quadraceps* spp. (*n* = 4, 1.1%), *Actornithophilus uniseriatus* (*n* = 2, 0.6%), *Nosopon chanabense* (*n* = 2, 0.6%), *Actornithophilus cornutus* (*n* = 1, 0.3%), *Cuclotogaster heterographus* (*n* = 1, 0.3%), *Falcolipeurus suturalis* (*n* = 1, 0.3%), *Laemobothrion atrum* (*n* = 1, 0.3%), *Colpocephalum gypsi* (*n* = 1, 0.3%), and *Rallicola cuspidatus* (*n* = 1, 0.3%). All of these species except six, i.e., *Trinoton* spp., *C*. *aquilinus*, *L*. *vulturis*, *L*. *maximum*, *C*. *impressum*, *C*. *turbinatum*, and *C*. *heterographus* are recorded for the first time from Iran. This study is the largest epidemiological study to date performed in the country. Data reported herein contribute to our knowledge about diversity of avian chewing lice from wild birds in Iran. In this paper, an updated checklist of louse species reported from Iran according to their avian hosts is presented.

## Introduction

Lice are small (0.35–11 mm long as adults), wingless, dorsoventrally flattened insects. They are obligatory, permanent ectoparasites of birds and mammals throughout the world which typically, parasitize individuals in small numbers and cause no apparent discomfort however, some of the lice can cause skin lesions and act as vectors or intermediate hosts of several bacteria, viruses and filarial parasites (1, 2). In addition, it has been shown that *Piagetiella titan* infesting white pelicans may invade the oral cavity causing erosions and petechial hemorrhages (3–5).

Lice (Insecta: Psocoptera: Phthiraptera) with about 5,000 known species, present on roughly 4,000 species of birds and 800 mammals, are categorized in four suborders (6). Species of the suborder Anoplura have adopted to suck blood from capillaries of mammals and ingest it, while Amblycera, Ischnocera, and Rhynchophthirina (formerly known as Mallophaga) have chewing mouth pieces, adapted to eat hairs and feathers, and sometimes also the skin and blood of birds and mammals (7). Avian chewing lice belong to one of two sub-orders: Amblycera, which occur on feathers and skin, or Ischnocera, which are more restricted to feathers (1). Most of the lice species are strongly associated with hosts, their phylogeny parallels that of hosts, sometimes with different speeds however, “host specificity” cannot be assumed (7, 8). Among different fields of wildlife parasitology, studying avian chewing lice is important as their epizootiology is largely associated with geographical distribution of their hosts.

Iran is a country in western Asia with a territory of 1,648,195 km^2^. It is the second largest country in the Middle East and the 17th largest in the world. In the country, 550 avian species are distributed which is almost equal to the richness of birds in Europe (9, 10). However, there is limited and scanty information about their parasites fauna specially the chewing lice (11–14) with several published in Persian language and presented in local congresses (15–19). Considering the scarcity of published records of lice in Iran, we aimed to gather new data and present an updated checklist of birds’ Phthiraptera occurring in the country.

## Materials and methods

**Figure 1:**
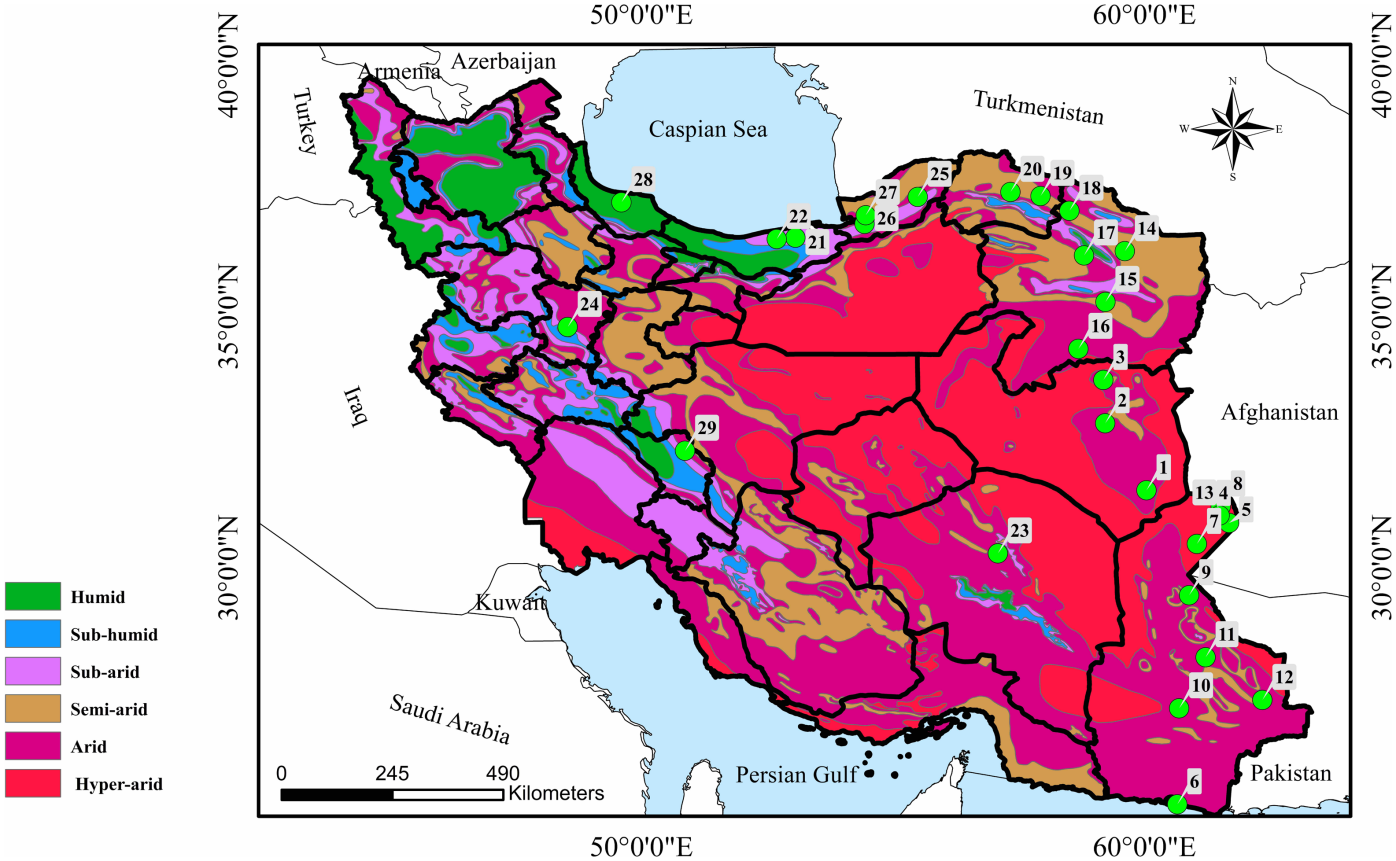
Map of Iran showing 29 sampling localities in nine provinces with number of birds examined in each city. (1) Nehbandan (*n* = 9), (2) Birjand (*n* = 10), (3) Qaen (*n* = 6), (4) Zabol (*n* = 93), (5) Zehak (*n* = 136), (6) Chabahar (*n* = 15), (7) Hamun (*n* = 59), (8) Hirmand (*n* = 29), (9) Zahedan (*n* = 26), (10) Iranshahr (*n* = 4), (11) Khash (*n* = 5), (12) Saravan (*n* = 5), (13) Nimruz (*n* = 27), (14) Mashhad (*n* = 12), (15) Torbat-e Heydariyeh (*n* = 4), (16) Gonabad (*n* = 2), (17) Neyshabur (*n* = 2), (18) Quchan (*n* = 3), (19) Shirvan (*n* = 5), (20) Bojnoord (*n* = 4), (21) Sari (*n* = 4), (22) Babol (*n* = 3), (23) Kerman (*n* = 73), (24) Hamedan (*n* = 54), (25) Kalaleh (*n* = 7), (26) Gorgan (*n* = 7), (27) Aqqala (*n* = 4), (28) Rasht (*n* = 2), and (29) Shahrekord (*n* = 2).

Between September 2019 and December 2023, totally 612 wild birds belonging 16 orders, 33 families, 60 genera, and 78 species from Hamedan (*n* = 54), nine different regions of Sistan-va-Baluchestan (*n* = 372), Kerman (*n* = 73), South Khorasan (*n* = 52), Mazandaran (*n* = 7), Chaharmahal-va-Bakhtiari (*n* = 2), Gilan (*n* = 2), Golestan (*n* = 18), North Khorasan (*n* = 9), and Razavi Khorasan (*n* = 23) were collected (Figure 1). The birds were euthanized by the Provincial Department of Environment because of general health failure or were found dead in the environment. The time lapse from death to examination of birds for lice infestation could not be estimated however, only fresh carcasses were examined. Individual birds were sent to Laboratory of Parasitology, Faculty of Veterinary Medicine, Bu-Ali Sina University in sealed plastic bags for examination or were examined in the field. The bird identifications were made using the reference book *Atlas of Birds of Iran* (9), and a standard examination for searching chewing lice was performed (20). The collected lice were placed in tubes containing 70% ethanol, cleared in 10% KOH for at least 1 day, mounted in Canada balsam on glass slides (21), and identified according to the original descriptions or keys (7, 22–35) using a Leica DM750 camera mounted trinocular microscope with Leica DFC295 application unit.

We also collected all the available information about chewing lice infesting birds in Iran. The databases and search engines employed for the literature review were Phthiraptera.info,^1^ PubMed,^2^ Google,^3^ Scientific Information Database of Iran,^4^ the collection of defended theses at all Iranian Universities,^5^ and the collection of proceedings of Iranian congresses.^6^ Valid names of the louse and bird species were obtained from Global Biodiversity Information Facility resources (36).

## Results

In total, 352 lice specimens including *Strigiphilus strigis n* = 55; 15.6% (Pontoppidan, 1763), *Falcolipeurus quadripustulatus n* = 41; 11.6% (Burmeister, 1838), *Craspedorrhynchus platystomus n* = 40; 11.3% (Burmeister, 1838), *Colpocephalum turbinatum n* = 36; 10.2% (Denny, 1842), *Laemobothrion maximum n* = 25; 7.1% (Scopoli, 1763), *Nosopon lucidum n* = 20; 5.6% (Rudow, 1869), *Degeeriella fulva n* = 18; 5.1% (Giebel, 1874), *Colpocephalum eucarenum n* = 16; 4.5% (Burmeister, 1838), *Laemobothrion vulturis n* = 15; 4.2% (Fabricius, 1775), *Anaticola crassicornis n* = 13; 3.7% (Scopoli, 1763), *Craspedorrhynchus aquilinus n* = 9; 2.5% (Denny, 1842), *Degeeriella fusca n* = 7; 2.0% (Denny, 1842), *Aegypoecus trigonoceps n* = 7; 2.0% (Giebel, 1874), *Quadraceps obscurus n* = 6; 1.7% (Burmeister, 1838), *Colpocephalum impressum n* = 6; 1.7% (Rudow, 1866), *Trinoton querquedulae n* = 6; 1.7% (Linnaeus, 1758), *Colpocephalum heterosoma n* = 5; 1.4% (Clay, 1951), *Colpocephalum nanum n* = 5; 1.4% (Piaget, 1890), *Lunaceps holophaeus n* = 4; 1.1% Burmeister, 1838, *Quadraceps* spp. (new species) *n* = 4; 1.1% (Clay and Meinertzhagen, 1939), *Actornithophilus uniseriatus n* = 2; 0.6% (Piaget, 1880), *Nosopon chanabense n* = 2; 0.6% (Ansari, 1951), *Actornithophilus cornutus n* = 1; 0.3% (Giebel, 1866), *Cuclotogaster heterographus n* = 1; 0.28% (Nitzsch, 1866), *Falcolipeurus suturalis n* = 1; 0.3% (Rudow, 1869), *Laemobothrion atrum n* = 1; 0.3% (Nitzsch, 1818), *Colpocephalum gypsi n* = 1; 0.3% (Eichler & Zlotorzycka, 1971), and *Rallicola cuspidatus n* = 1; 0.3% (Scopoli, 1763) were collected from 58/612 birds (9.5%). Collected lice specimens belonged to 31 species from the families Philopteridae, Menoponidae, and Laemobothriidae. All of the identified lice species except *C*. *aquilinus*, *L*. *vulturis*, *L*. *maximum*, *C*. *impressum*, *C*. *turbinatum*, and *C*. *heterographus* are recorded for the first time from Iran (Table 1).

**Table 1:** Distribution of louse species of wild birds in some regions of Iran (September 2019 and December 2023) according to their host bird species.

	Host information			Parasite information									
*n* birds examined	Host bird taxonomy	Host scientific name	Host vernacular name	Louse species	Suborder	Family	City	*n* total/infested	Louse prevalence (*n*)				
									Male	Female	Nymph	Damaged	Total
	**ACCIPITRIFORMES**												
	**Accipitridae**												
1		*Accipiter badius* (Gmelin, 1788)	Shikra	-	-	-	Zahedan	0/1					
8		*Accipiter nisus* (Linnaeus, 1758)	Eurasian sparrowhawk	-	-	-	Hamun	0/2					
							Hamedan	0/5					
							Zabol	0/1					
1		*Aegypius monachus* (Linnaeus, 1758)	Cinereous vulture	-	-	-	Zahedan	0/1					
4		*Aquila chrysaetos* (Linnaeus, 1758)	Golden eagle	*Craspedorrhynchus aquilinus* (Denny, 1842)	Ischnocera	Philopteridae	Hamedan	3/3	5	4	0	0	9
							Kerman	0/1					
1		*Aquila heliaca* (Savigny, 1809)	Asian imperial eagle	*Laemobothrion maximum* (Scopoli, 1763)	Amblycera	Laemobothriidae	Hamedan	1/1	0	0	3	0	3
3		*Aquila nipalensis* (Hodgson, 1833)	Steppe eagle	*Laemobothrion maximum* (Scopoli, 1763)	Amblycera	Laemobothriidae	Hamedan	2/3	2	4	0	0	6
				*Laemobothrion vulturis* (Fabricius, 1775)				1/3	0	2	2	0	4
				*Colpocephalum impressum* Rudow, 1866		Menoponidae		1/3	0	1	0	0	1
				*Craspedorrhynchus aquilinus* (Denny, 1842)	Ischnocera	Philopteridae		1/3	0	1	0	0	1
				*Falcolipeurus suturalis* (Rudow, 1869)				1/3	0	1	0	0	1
1		*Aquila rapax* (Temminck, 1828)	Tawny eagle	*Laemobothrion vulturis* (Fabricius, 1775)	Amblycera	Laemobothriidae	Hamedan	1/1	1	0	0	0	1
				*Colpocephalum impressum* (Rudow, 1866)		Menoponidae		1/1	4	0	1	0	5
				*Nosopon chanabense* (Ansari, 1951)				1/1	0	2	0	0	2
14		*Buteo buteo* (Linnaeus, 1758)	Buzzard	*Degeeriella fulva* (Giebel, 1874)	Ischnocera	Philopteridae	Hamedan	1/14	10	8	0	0	18
				*Degeeriella fusca* (Denny, 1842)				1/14	2	4	1	0	7
				*Cuclotogaster heterographus* (Nitzsch, 1866)				1/14	1	0	0	0	1
				*Craspedorrhynchus platystomus* (Burmeister, 1838)				2/14	18	19	3	0	40
				*Colpocephalum nanum* (Piaget, 1890)	Amblycera	Menoponidae		1/14	0	2	2	1	5
				*Colpocephalum turbinatum* (Denny, 1842)				1/14	24	12	0	0	36
				*Laemobothrion maximum* (Scopoli, 1763)		Laemobothriidae	Kerman	1/12	3	3	6	0	12
3		*Buteo rufinus* (Cretzschmar, 1829)	The long-legged buzzard	*Laemobothrion maximum* (Scopoli, 1763)	Amblycera	Laemobothriidae	Kerman	1/1	1	1	1	0	3
							Zabol	0/1					
							Zahedan	0/1					
3		*Circus aeruginosus* (Linnaeus, 1758)	Eurasian marsh-harrier	*Nosopon lucidum* (Rudow, 1869)	Amblycera	Menoponidae	Hamedan	1/3	6	12	2	0	20
2		*Circaetus gallicus* (Gmelin, 1788)	Short-toed snake eagle	-	-	-	Zahedan	0/2					
4		*Gyps fulvus* (Hablizl, 1783)	Griffon vulture	*Laemobothrion vulturis* (Fabricius, 1775)	Amblycera	Laemobothriidae	Hamedan	2/4	1	5	4	0	10
				*Colpocephalum gypsi* (Eichler & Zlotorzycka, 1971)		Menoponidae	Zabol	1/4	1	0	0	0	1
				*Colpocephalum* spp.				1/4	0	0	0	1	1
				*Falcolipeurus quadripustulatus* (Burmeister, 1838)	Ischnocera	Philopteridae		2/4	23	18	0	0	41
				*Aegypoecus trigonoceps* (Giebel, 1874)			Kerman	1/4	3	3	1	0	7
	**ANSERIFORMES**												
	**Anatidae**												
6		*Aythya ferina* (Linnaeus, 1758)	Common pochard	-	-	-	Zehak	0/3					
							Zabol	0/1					
							Chabahar	0/2					
10		*Anas crecca* (Linnaeus, 1758)	Common Teal	*Trinoton querquedulae* (Linnaeus, 1758)	Amblycera	Menoponidae	Zehak	5/8	0	3	0	0	3
				*Anaticola crassicornis* (Scopoli, 1763)	Ischnocera	Philopteridae	Chabahar	0/2	3	5	1	0	9
7		*Anas platyrhynchos* (Linnaeus, 1758)	Mallard	*Anaticola crassicornis* (Scopoli, 1763)	Ischnocera	Philopteridae	Zehak	0/1	3	1	0	0	4
														Zabol	2/2
							*Trinoton querquedulae* (Linnaeus, 1758)	Amblycera						Menoponidae	Hamedan	1/1	0	1	0	0	1
				Chabahar	0/3																
				1		*Anas penelope* (Linnaeus, 1758)	Eurasian wigeon	*Laemobothrion* spp.	Amblycera	Laemobothriidae	Zabol	1/1	0	0	1	0	1
9		*Spatula clypeata* (Linnaeus, 1758)	Northern shoveler	*Pectinopygus* spp.	Ischnocera	Philopteridae	Zabol	1/3	0	1	0	0	1
							Zehak						
							Chabahar						
1		*Mergus merganser* (Linnaeus, 1758)	Common merganser	-	-	-	Zabol	0/1					
2		*Spatula querquedula* (Linnaeus, 1758)	Garganey	*Trinoton querquedulae* (Linnaeus, 1758)	Amblycera	Menoponidae	Zehak	1/2	1	0	0	0	1
2		*Tadorna tadorna* (Linnaeus, 1758)	Common shelduck	-	-	-	Zehak	0/2					
	**BUCEROTIFORMES**												
3	**Upupidae**	*Upupa epops* (Linnaeus, 1758)	Eurasian hoopoe	-	-	-	Nehbandan	0/1					
							Birjand	0/1					
							Zabol	0/1					
	**CHARADRIIFORMES**												
	**Recurvirostridae**												
4		*Himantopus himantopus* (Linnaeus, 1758)	Black-winged stilt	*Actornithophilus uniseriatus* (Piaget, 1880)	Amblycera	Menoponidae	Zehak	4/4	1	0	1	0	2
				*Quadraceps* spp.	Ischnocera	Philopteridae			3	0	1	0	4
	**Scolopacidae**												
3		*Philomachus pugnax* (Linnaeus, 1758)	Ruff	*Lunaceps holophaeus* (Burmeister, 1838)	Mallophaga	Philopteridae	Zehak	3/3	2	2	0	0	4
				*Actornithophilus cornutus* (Giebel, 1866)	Amblycera	Menoponidae			1	0	0	0	1
2		*Tringa stagnatilis* (Bechstein, 1803)	Marsh sandpiper	*Quadraceps obscurus* (Burmeister, 1838)	Ischnocera	Philopteridae	Zehak	2/2	3	3	0	0	6
14		*Phalaropus lobatus* (Linnaeus, 1758)	Red-necked phalarope	*-*	-	-	Zehak	0/14					
	**Laridae**												
17		*Sterna repressa* (Hartert, 1916)	White-cheeked tern	-	-	-	Zehak	0/7					
							Zabol	0/4					
							Hamun	0/6					
	**CAPRIMULGIFORMES**												
	**Caprimulgidae**												
8		*Caprimulgus aegyptius* (Lichtenstein, 1823)	Egyptian nightjar	-	-	-	Hamun	0/3					
							Zabol	0/1					
							Nimruz	0/2					
							Zehak	0/2					
	**COLUMBIFORMES**												
	**Columbidae**												
22		*Streptopelia decaocto* (Frivaldszky, 1838)	Eurasian collared dove	-	-	-	Hirmand	0/4					
							Zabol	0/4					
							Nimruz	0/3					
							Zehak	0/6					
							Hamun	0/5					
36		*Spilopelia senegalensis* (Linnaeus, 1766)	Laughing dove	-	-	-	Hirmand	0/1					
							Zabol	0/8					
							Nimruz	0/2					
							Zehak	0/5					
							Sari	0/1					
							Kerman	0/2					
							Zahedan	0/2					
							Mashhad	0/3					
							Kalaleh	0/2					
							Gorgan	0/2					
							Nehbandan	0/1					
							Birjand	0/1					
							Hamun	0/6					
	**FALCONIFORMES**												
	**Falconidae**												
46		*Falco tinnunculus* (Linnaeus, 1758)	Kestrel	*Laemobothrion maximum* (Scopoli, 1763)	Amblycera	Laemobothriidae	Hamedan	1/12	0	0	1	0	1
							Kerman	0/26					
							Zabol	0/4					
							Zahedan	0/4					
4		*Falco cherrug* (Gray, 1834)	Saker falcon	-	-	-	Kerman	0/4					
1		*Falco naumanni* (Fleischer, 1818)	Lesser kestrel	-	-	-	Zabol	0/1					
2		*Falco peregrinus* subsp. *pelegrinoides* (Temminck, 1829)	Barbary falcon	-	-	-	Kerman	0/2					
	**GALLIFORMES**												
	**Phasianidae**												
22		*Ammoperdix griseogularis* (Brandt, 1843)	See-see partridge	-	-	-	Zahedan	0/8					
							Nehbandan	0/3					
							Iranshahr	0/1					
							Khash	0/3					
							Chabahar	0/1					
							Birjand	0/1					
							Qaen	0/1					
							Torbat-Heidarie	0/2					
							Gonaabaad	0/1					
							Saravan	0/1					
11		*Alectoris chukar* (Gray, 1830)	Chukar	-	-	-	Iranshahr	0/3					
							Saravan	0/4					
							Chabahar	0/1					
							Zahedan	0/3					
15		*Coturnix coturnix* (Linnaeus, 1758)	Common quail	-	-	-	Kerman	0/14					
							Zabol	0/1					
5		*Francolinus francolinus* (Linnaeus, 1766)	Black francolin	-	-	-	Hirmand	0/1					
							Zehak	0/1					
							Hamun	0/3					
	**GRUIFORMES**												
	**Rallidae**												
1		*Rallus aquaticus* (Linnaeus, 1758)	Water rail	*Rallicola cuspidatus* (Scopoli, 1763)	Ischnocera	Philopteridae	Hamedan	1/1	1	0	0	0	1
18		*Fulica atra* (Linnaeus, 1758)	Coot	*Laemobothrion* (*Eulaemobothrion*) *atrum* (Nitzsch, 1818)	Amblycera	Laemobothriidae	Zehak	0/12						Zabol	1/3	0	1	0	0	1	Hamun	0/2						Nimruz	0/1					
														**OTIDIFORMES**												
								**Otididae**												
							1		*Chlamydotis macqueenii* (Gray, 1832)	MacQueen’s bustard	-	-	-	Kerman	0/1					
								**PASSERIFORMES**												
4	**Acrocephalidae**	*Acrocephalus scirpaceus* (Hermann, 1804)	Eurasian reed warbler	*-*	-	-	Zehak	0/2					
							Zabol	0/2					
	**Alaudidae**												
5		*Alaemon alaudipes* (Desfontaines, 1789)	Greater hoopoe-lark				Hirmand	0/2					
							Zabol	0/2					
							Zehak	0/1					
11		*Alauda arvensis* (Linnaeus, 1758)	Eurasian skylark	*-*	-	-	Hamun	0/2					
							Zehak	0/1					
							Hirmand	0/2					
							Zabol	0/4					
							Nimruz	0/2					
35		*Galerida cristata* (Linnaeus, 1758)	Crested lark	*-*	-	-	Hamun	0/4					
														Zabol	0/4
							Hirmand	0/4					
							Birjand	0/2					
							Neishaboor	0/1					
							Quchan	0/1					
							Torbat-Heidarie	0/1					
							Gonaabaad	0/1					
							Gorgan	0/2					
							Shirvan	0/2					
							Bojnurd	0/2					
							Sari	0/2					
							Qaen	0/1					
							Nimruz	0/4					
							Zehak	0/4					
							4							*Melanocorypha calandra* (Linnaeus, 1766)	Calandra lark	-	-	-	Mashhad	0/4					
	**Cisticolidae**												
7		*Prinia gracilis* (Lichtenstein, 1823)	Graceful prinia	*-*	-	-	Zehak	0/3					
							Hamun	0/2					
							Zabol	0/2					
	**Fringillidae**												
5		*Serinus pusillus* (Pallas, 1811)	Red-fronted serin	*-*	-	-	Kerman	0/5					
5		*Carduelis carduelis* (Linnaeus, 1758)	European goldfinch	*-*	-	-	Mashhad	0/1					
							Birjand	0/1					
							Gorgan	0/1					
							Kalaleh	0/1					
							Kerman	0/1					
11		*Rhodospiza obsoleta* (Lichtenstein, 1823)	Desert finch	*-*	-	-	Nehbandan	0/2					
							Qaen	0/3					
							Mashhad	0/3					
							Bojnurd	0/1					
							Birjand	0/1					
							Torbat-Heidarieh	0/1					
	**Hirundinidae**												
7		*Hirundo rustica* (Linnaeus, 1758)	Barn swallow				Hirmand	0/2					
							Hamun	0/1					
							Zehak	0/4					
	**Passeridae**												
19		*Passer hispaniolensis* (Temminck, 1820)	Spanish sparrow	*-*	-	-	Nimruz	0/3					
							Zabol	0/5					
							Zehak	0/6					
							Hamun	0/2					
							Hirmand	0/3					
23		*Passer domesticus* (Linnaeus, 1758)	House sparrow	*-*	-	-	Shirvan	0/3					
							Gorgan	0/1					
							Kalaleh	0/4					
							Aqqala	0/4					
							Rasht	0/2					
							Sari	0/1					
							Babol	0/3					
							Birjand	0/3					
							Nehbandan	0/1					
							Quchan	0/1					
30		*Passer montanus* (Linnaeus, 1758)	Eurasian tree sparrow	*-*	-	-	Hamun	0/7					
							Zabol	0/9					
							Hirmand	0/3					
							Zehak	0/5					
							Nimruz	0/6					
	**Pycnonotidae**												
21		*Pycnonotus leucotis* (Gould, 1836)	White-eared bulbul	*-*	-	-	Hamun	0/4					
							Zabol	0/6					
							Hirmand	0/4					
							Zehak	0/5					
							Nimruz	0/2					
	**Laniidae**												
4		*Lanius phoenicuroides* (Schalow, 1875)	Red-tailed shrike	*-*	-	-	Hirmand	0/1					
							Hamun	0/1					
							Zehak	0/1					
							Zabol	0/1					
	**Leiothrichidae**												
14		*Turdoides caudata* (Dumont, 1823)	Common babbler	*-*	-	-	Hamun	0/4					
							Zabol	0/3					
							Hirmand	0/2					
							Zehak	0/3					
							Nimruz	0/2					
	**Motacillidae**												
11		*Motacilla alba* (Linnaeus, 1758)	White wagtail	*-*	-	-	Gorgan	0/1					
							Quchan	0/1					
							Bojnurd	0/1					
							Neishaboor	0/1					
							Mashhad	0/1					
							Zabol	0/1					
							Qaen	0/1					
							Nehbandan	0/1					
							Zehak	0/2					
							Hamun	0/1					
	**Muscicapidae**												
7		*Oenanthe albonigra* (Hume, 1872)	Hume’s wheatear	*-*	-	-	Zehak	0/2					
							Hamun	0/1					
							Zabol	0/4					
1		*Cercotrichas galactotes* (Temminck, 1820)	Rufous-tailed scrub robin	-	-	-	Zabol	0/1					
	**Scotocercidae**												
5		*Scotocerca inquieta* (Cretzschmar, 1830)	Streaked scrub warbler	*-*	-	-	Zehak	0/2					
							Hamun	0/1					
							Zabol	0/2					
	**PELECANIFORMES**												
	**Ardeidae**												
6		*Ardea cinerea* (Linnaeus, 1758)	Gray heron	*-*	-	-	Hamedan	0/1					
							Zehak	0/5					
5		*Ardea alba* (Linnaeus, 1758)	Great egret				Zehak	0/3					
							Hamun	0/2					
1		*Botaurus stellaris* (Linnaeus, 1758)	Great Bittern (Eurasian bittern)	*-*	-	-	Hamedan	0/1					
2		*Ixobrychus minutus* (Linnaeus, 1766)	Little bittern	*-*	-	-	Zahedan	0/2					
	**Pelecanidae**												
4		*Pelecanus crispus* (Bruch, 1832)	Dalmatian pelican	*Colpocephalum eucarenum* Burmeister, 1838	Amblycera	Menoponidae	Zehak	0/2	6	5	5	0	16
							Zabol	1/2					
	**PHOENICOPTERIFORMES**												
	**Phoenicopteridae**												
3		*Phoenicopterus ruber* (Linnaeus, 1758)	American flamingo	*Colpocephalum heterosoma* Piaget, 1880, small specimen (Clay, 1951)	Amblycera	Menoponidae	Zabol	1/1	1	3	0	0	4
				*Colpocephalum heterosoma* Piaget, 1880, large specimen			Zehak	0/2	1	0	0	0	1
	**PODICIPEDIFORMES**												
	**Podicipedidae**												
9		*Podiceps cristatus* (Linnaeus, 1758)	Great crested grebe	-	-	-	Zehak	0/6					
							Zabol	0/3					
	**PTEROCLIDIFORMES**												
	**Pteroclidae**												
1		*Pterocles orientalis* (Linnaeus, 1758)	Black-bellied Sandgrouse	*-*	-	-	Hamedan	0/1					
	**PICIFORMES**												
	**Picidae**												
1		*Dendrocopos syriacus* (Hemprich & Ehrenberg, 1833)	Syrian woodpecker	*-*	-	-	Hamedan	0/1					
	**SULIFORMES**												
	**Phalacrocoracidae**												
7		*Phalacrocorax carbo* (Linnaeus, 1758)	Great cormorant	-	-	-	Zabol	0/2					
							Zehak	0/2					
							Chabahar	0/3					
	**STRIGIFORMES**												
	**Strigidae**												
2		*Asio otus* (Linnaeus, 1758)	Long-eared owl	*Strigiphilus* sp.	Ischnocera	Philopteridae	Hamedan	1/2	0	1	0	0	1
9		*Athene noctua* (Scopoli, 1769)	Little owl	*-*	-	-	Hamedan	0/1					
							Kerman	0/4					
							Shahrekord	0/2					
							Khash	0/2					
8		*Bubo bubo* (Linnaeus, 1758)	Eagle owl	*Strigiphilus strigis* (Pontoppidan, 1763)	Ischnocera	Philopteridae	Hamedan	2/2	26	29	0	0	55
							Zahedan	0/1					
							Zabol	0/1					
							Kerman	0/4					
1		*Otus scops* (Linnaeus, 1758)	European scops owl	*-*	-	-	Hamedan	0/1					
2		*Otus brucei (Hume*, *1873)*	Pallid scops owl	*-*	-	-	Kerman	0/2					
	**Tytonidae**												
4		*Tyto alba* (Scopoli, 1769)	Barn owl	*-*	-	-	Hamedan	0/1					
							Kerman	0/2					
							Zahedan	0/1					
Total 612								58	157	157	35	2	352

^a^according to International Union for Conservation of Nature (IUCN) Red List of Threatened Species (www.iucnredlist.org). Names of orders are capitalized, and names of families are showed in bold.

Number of lice specimens collected from examined birds ranged from 1 to 55, the latter was a *Bubo bubo* Linnaeus, 1758. Mixed lice infestation was found in 11 birds, i.e., in one *Philomachus pugnax* (Linnaeus, 1758), two *Himantopus himantopus* Linnaeus, 1758, two *Anas crecca* Linnaeus, 1758, one *Aquila nipalensis* Hodgson, 1833, one *Aquila rapax* Temminck, 1828, two *Gyps fulvus* Hablitz, 1783, and two *Buteo buteo* Linnaeus, 1758. Photomicrographs of examined lice specimens are presented in Figures 2–14.

**Figure 2:**
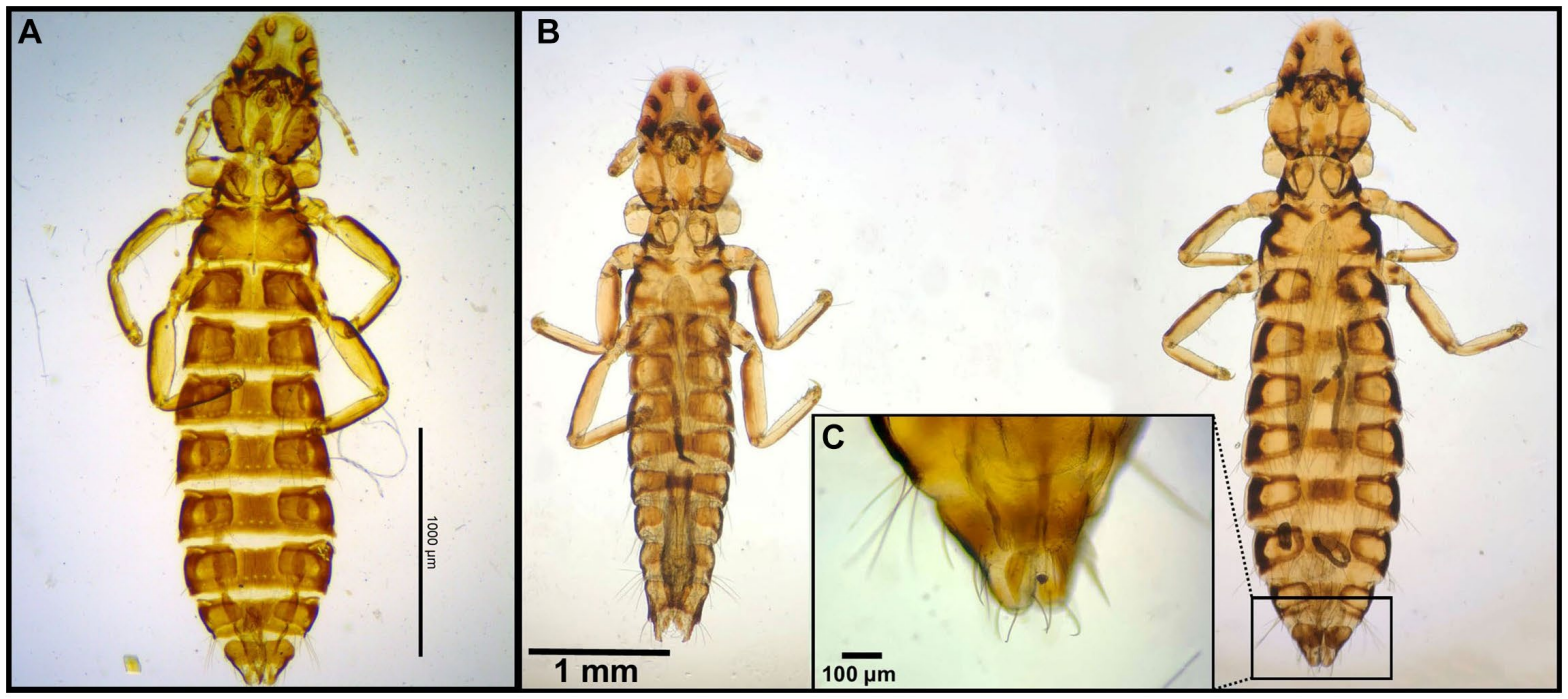
Chewing lice identified in this study part I: **(A)** *Falcolipeurus suturalis* ♀; **(B)** *Falcolipeurus quadripustulatus* left ♂, right ♀; and **(C)** ♀ posterior end. The map was drawn by using ArcGIS software version 10.3 (https://enterprise.arcgis.com/en/portal/).

**Figure 3:**
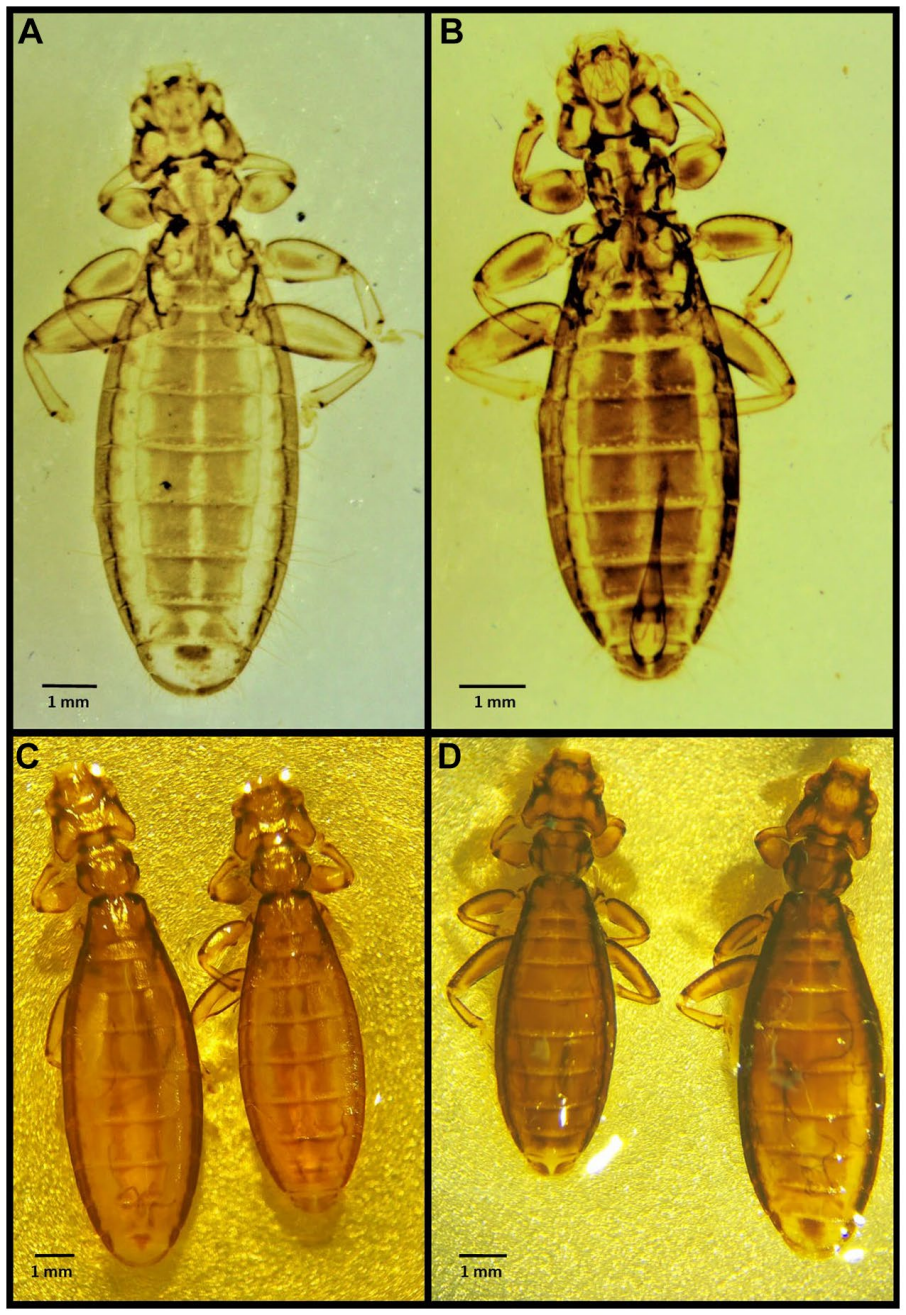
Chewing lice identified in this study part II: **(A)** *Laemobothrion maximum* ♀; (**B**) *Laemobothrion maximum* ♂; **(C)** Stereomicroscope picture of *Laemobothrion vulturis* left ♀, right ♂; and **(D)** Stereomicroscope picture of *Laemobothrion maximum* left ♂, right ♀.

**Figure 4:**
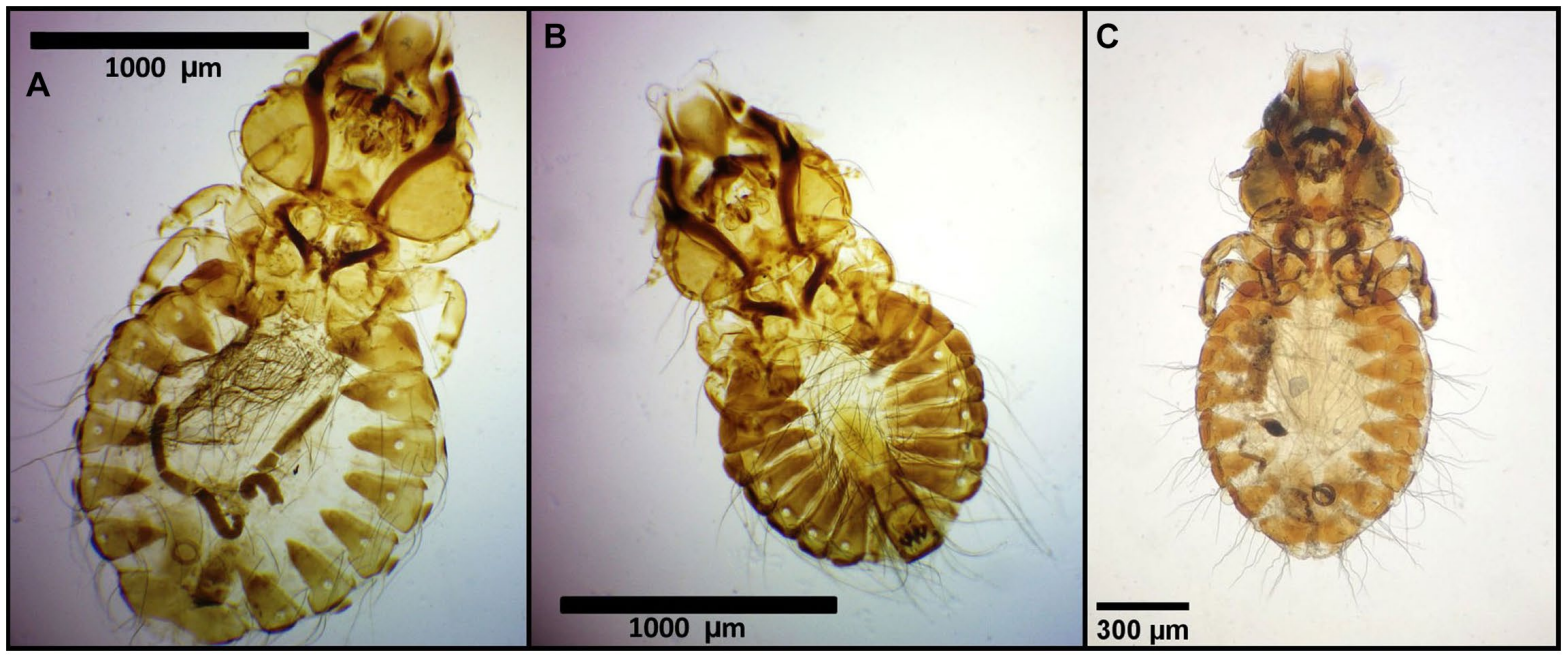
Chewing lice identified in this study part III: **(A)** *Craspedorrhynchus aquilinus* ♀, **(B)** *Craspedorrhynchus aquilinus* ♂; and **(C)** *Craspedorrhynchus platystomus* ♀.

**Figure 5:**
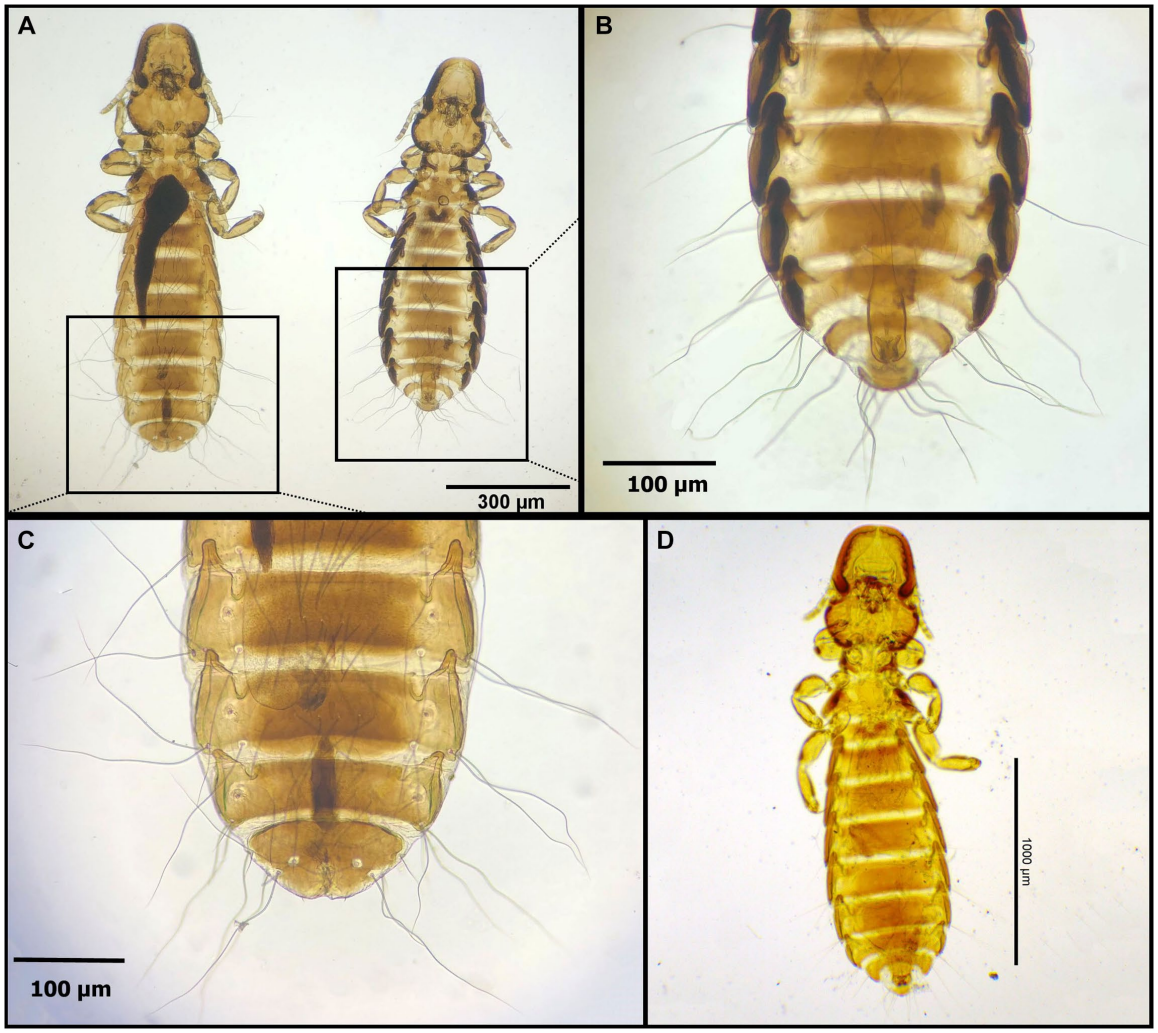
Chewing lice identified in this study part IV: **(A–C)** *Degeeriella fusca*, **(A)** left ♀, right ♂; **(B)** ♂, posterior part of the abdomen; **(C)** ♀, posterior part of the abdomen; and **(D)** *Degeeriella fulva* ♂.

**Figure 6:**
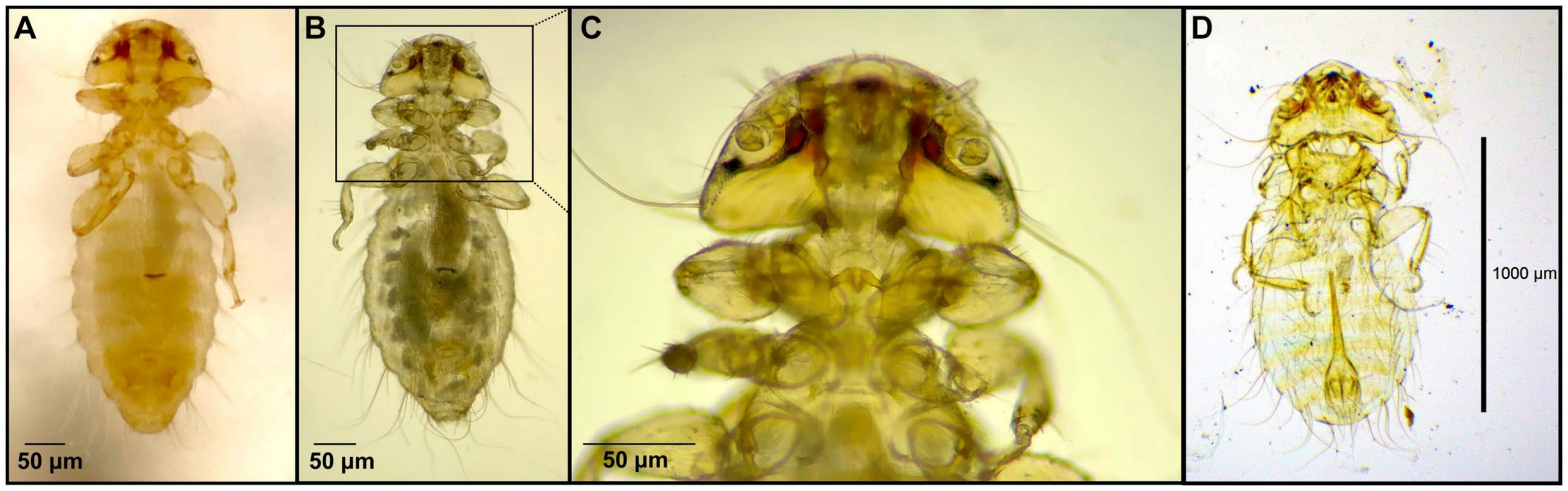
Chewing lice identified in this study part V: **(A–C)** *Nosopon chanabense* ♀; **(A)** Stereomicroscope picture; **(B)** Light microscope picture; **(C)** Head; and **(D)** *Nosopon lucidum* ♂.

**Figure 7:**
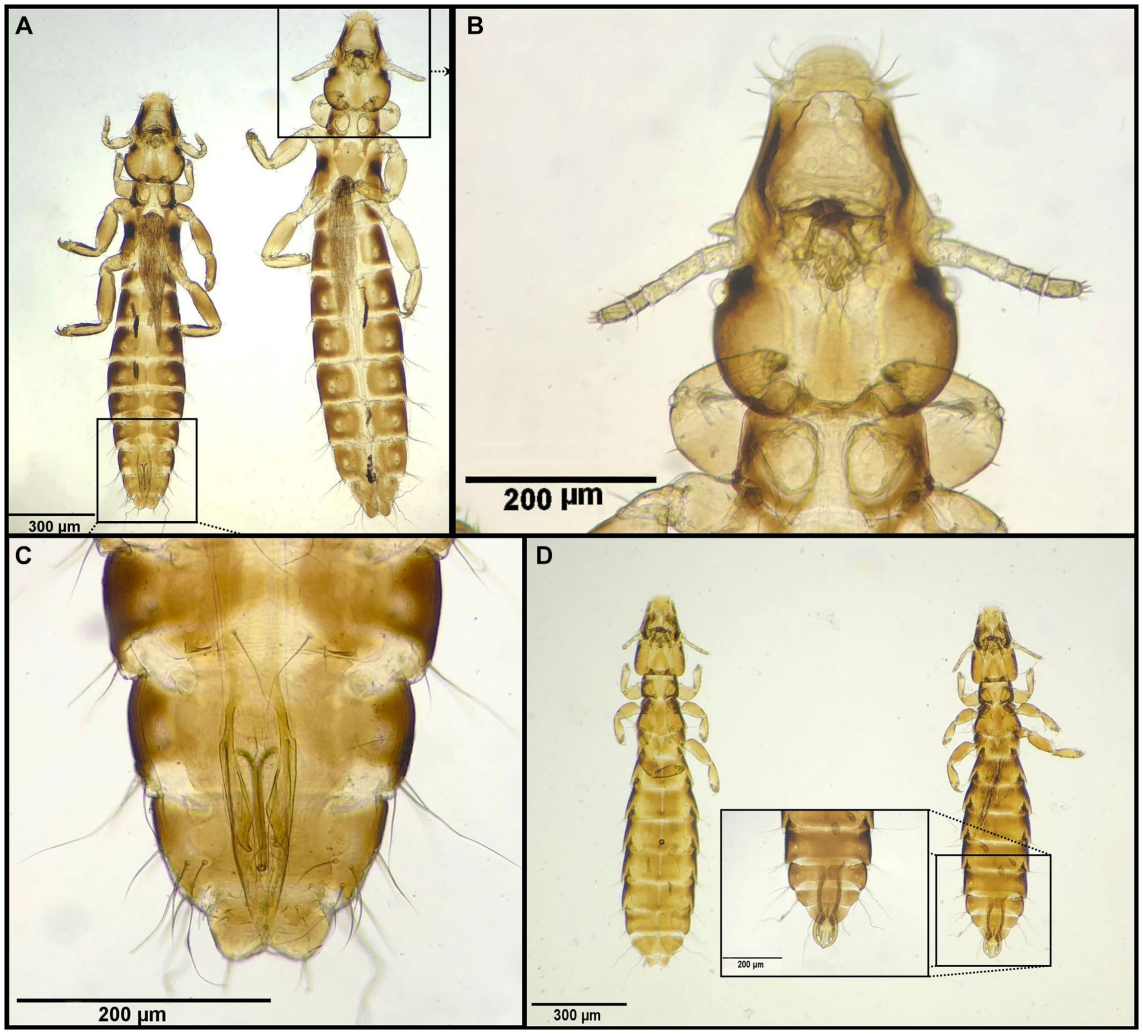
Chewing lice identified in this study part VI: **(A–C)** *Anaticola crassicornis* left ♂, right ♀; **(B)** ♀, Head; **(C)** ♂, posterior end; and **(D)** *Quadraceps obscurus* left ♀, right ♂.

**Figure 8:**
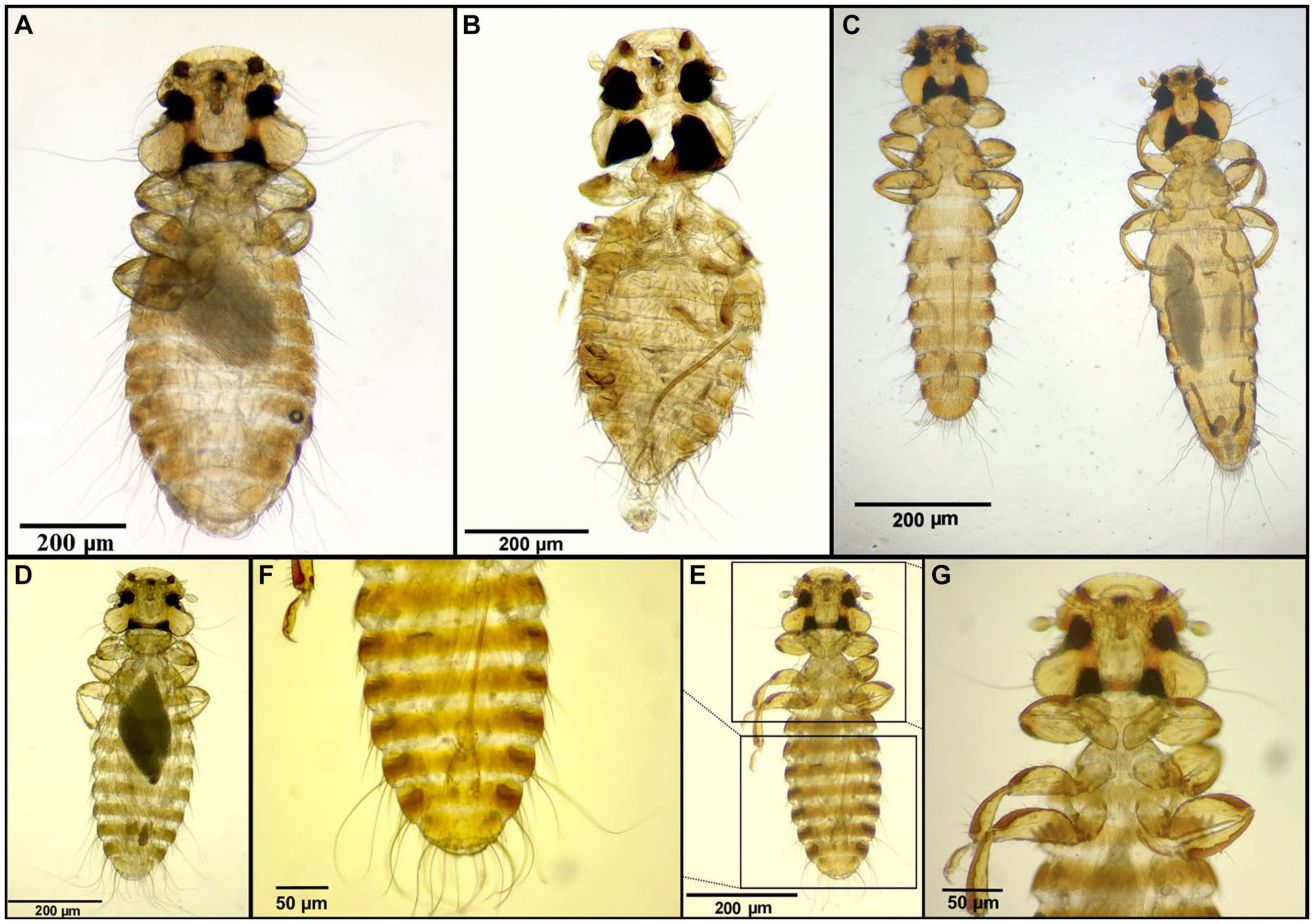
Chewing lice identified in this study part VII: **(A)** *Colpocephalum nanum* ♀; **(B)** *Colpocephalum gypsi* ♂; **(C)** *Colpocephalum eucarenum* left ♂, right ♀; **(D–G)** *Colpocephalum impressum*; **(D)** ♀; **(E)** ♂; **(F)** ♂, posterior end; and **(G)** ♂, head.

**Figure 9:**
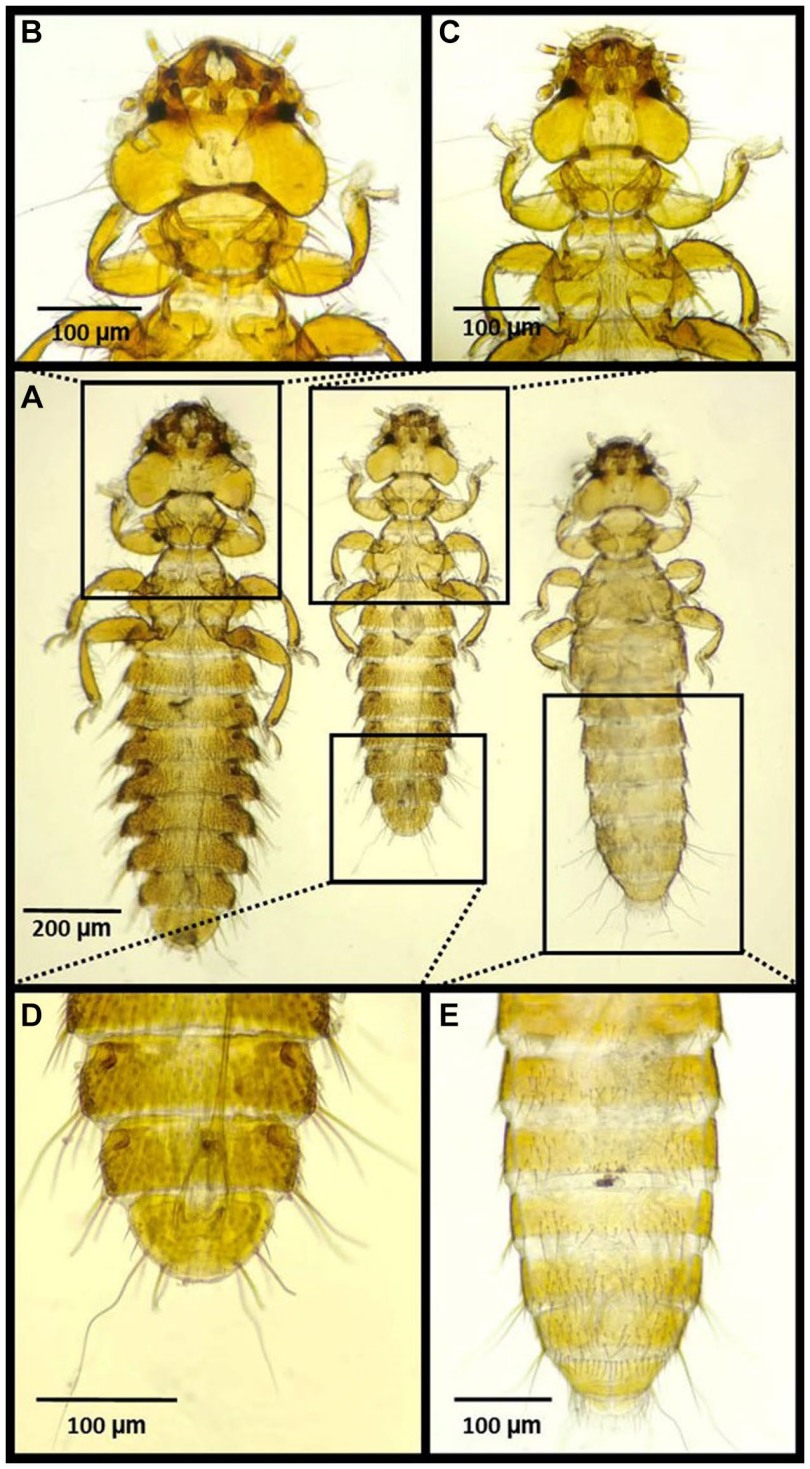
Chewing lice identified in this study part VIII: **(A–E)** *Colpocephalum heterosoma*; **(B)** Head of large specimen ♂; **(C)** Head of small specimen, ♂; **(D)** Posterior end of small specimen ♂, and **(E)** Posterior end of small specimen ♀.

**Figure 10:**
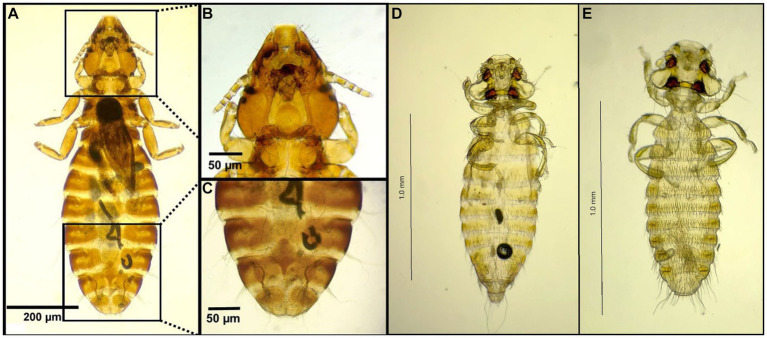
Chewing lice identified in this study part IX: **(A–C)** *Pectinopygus* spp. ♀; **(B)** Head; **(C)** Posterior end; **(D)** *Colpocephalum turbinatum* ♀; and **(E)** *Colpocephalum turbinatum* ♂.

**Figure 11:**
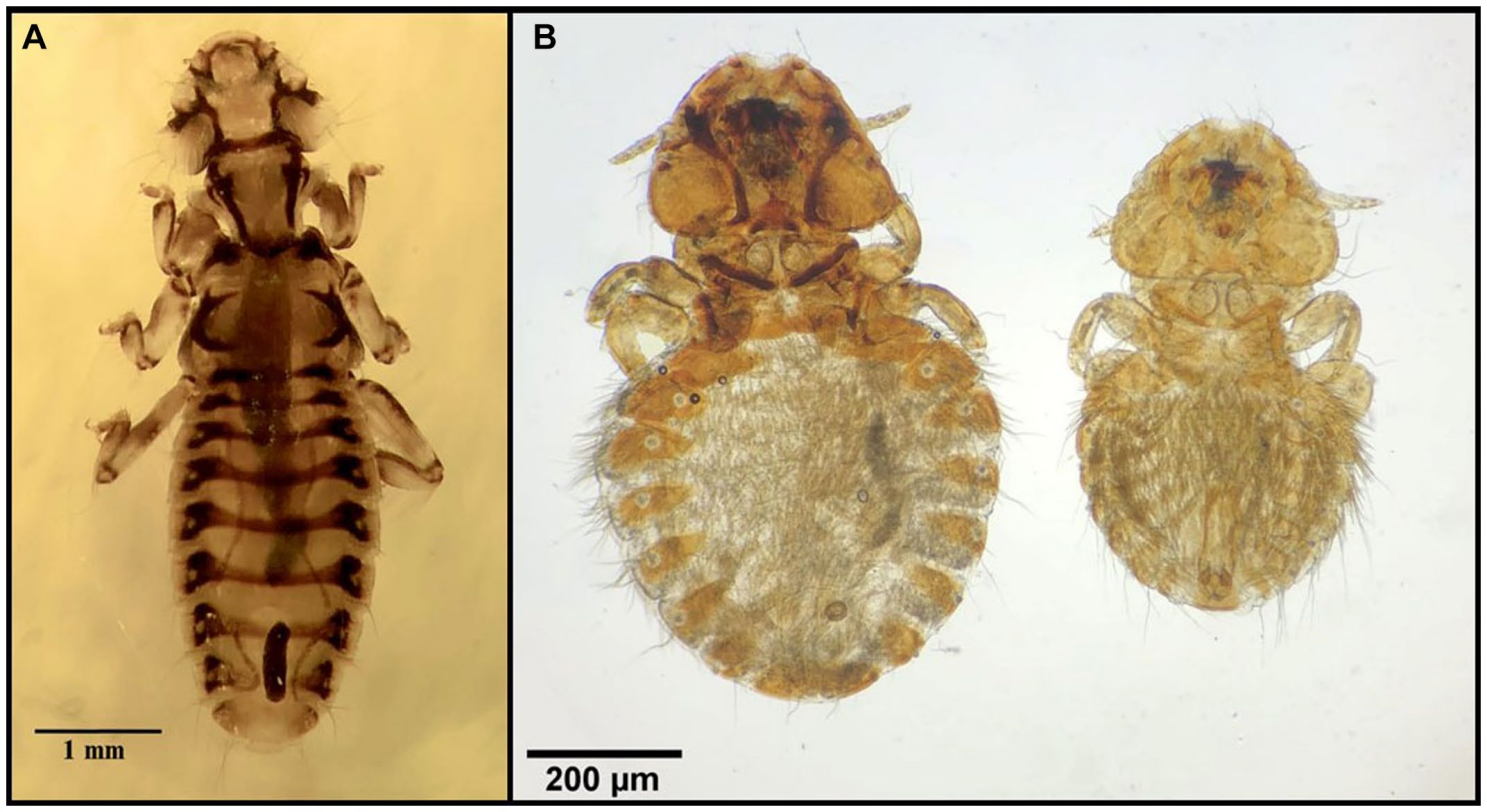
Chewing lice identified in this study part X: **(A)** *Trinoton querquedulae* ♀; **(B)** *Aegypoecus trigonoceps* left ♀, right ♂.

**Figure 12:**
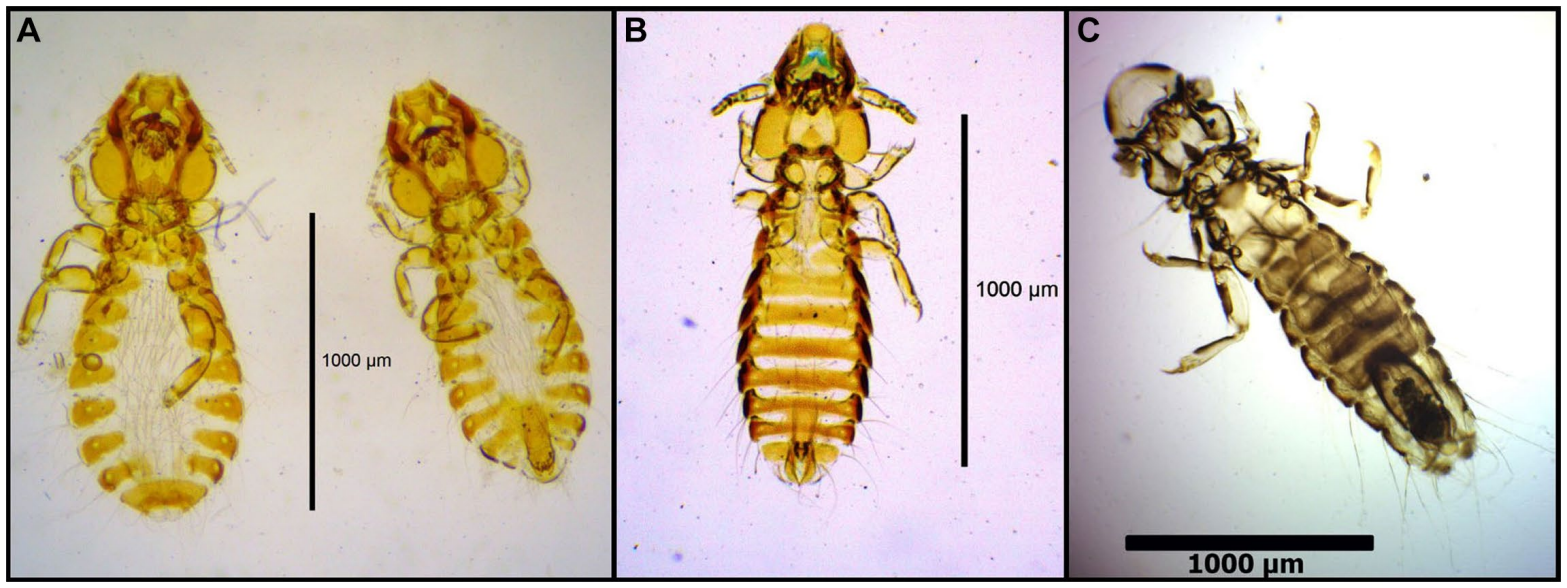
Chewing lice identified in this study part XI: **(A)** *Strigiphilus strigis* left ♀, right ♂; **(B)** *Rallicola cuspidatus* ♂; and **(C)** *Cuclotogaster heterographus* ♂.

**Figure 13:**
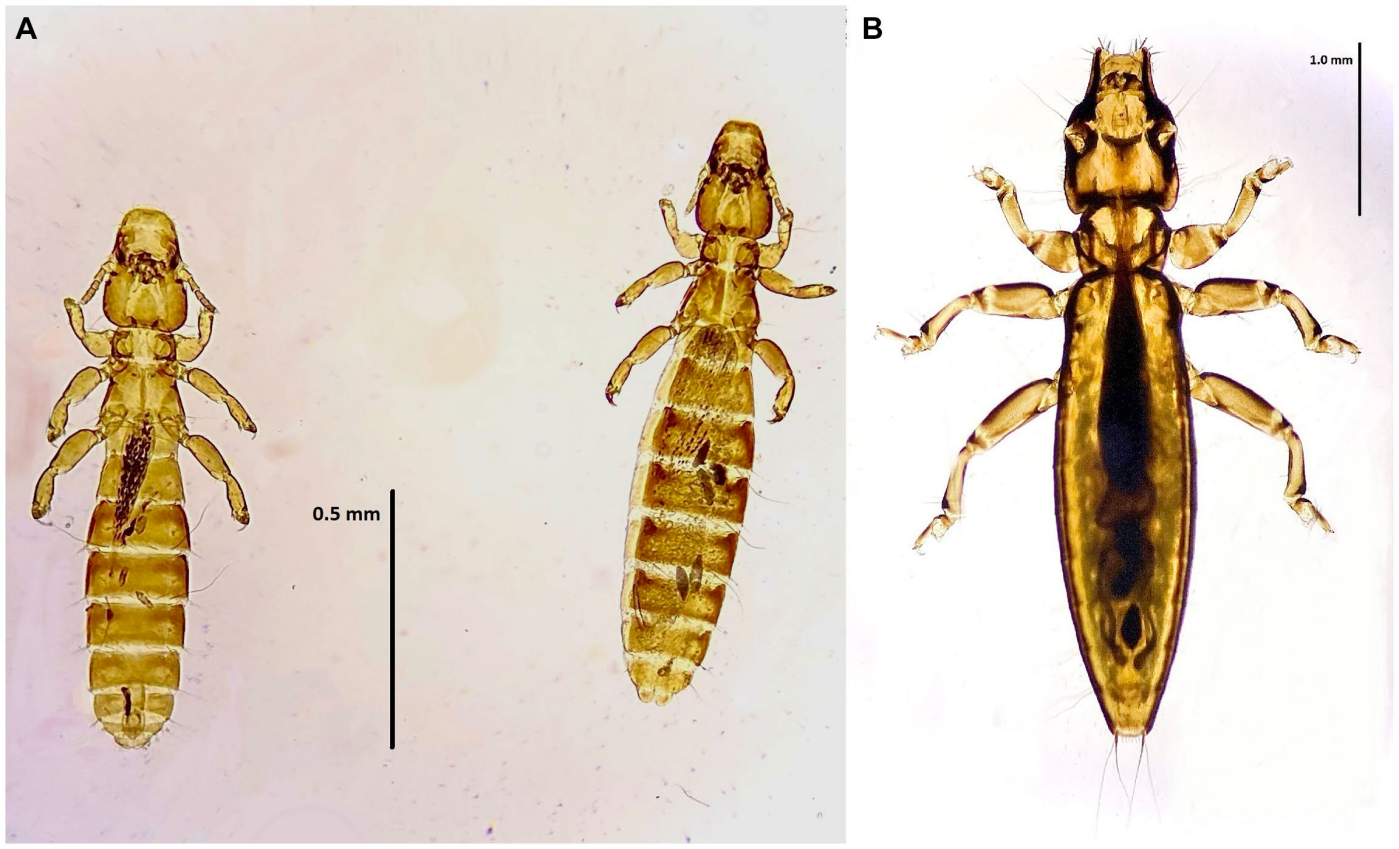
Chewing lice identified in this study part XII: **(A)** *Lunaceps holophaeus* left ♂, right ♀; **(B)** *Laemobothrion* spp. nymph.

**Figure 14:**
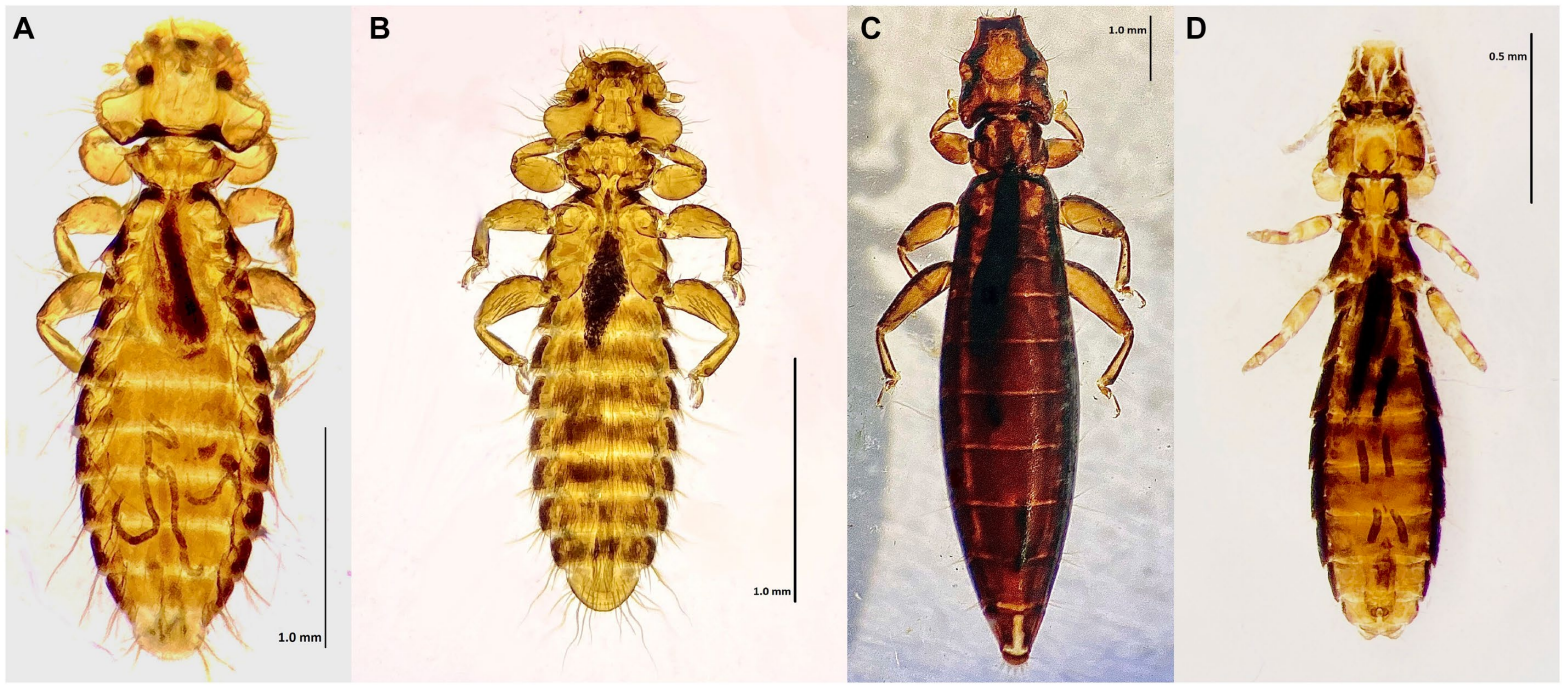
Chewing lice identified in this study part XIII: **(A)** *Actornithophilus uniseriatus* ♂; **(B)** *Actornithophilus cornutus* ♂; **(C)** *Lamobothrion atrum* ♀; and **(D)** *Quadraceps* spp. ♂.

Identification of few specimens could not be performed to species level including one damaged female *Strigiphilus* sp. collected from *Asio otus* Linnaeus, 1758, one female *Pectinopygus* spp. collected from *Anas clypeata* Linnaeus, 1758 which is an unusual host and possibly was a contamination, one *Laemobothrion* spp. nymph, and four *Quadraceps* spp. (Figures 10A–C, 13B, 14D). In addition, nits collected from one *Falco tinnunculus* Linnaeus, 1758 could not be identified.

In Supplementary Table 1, the information in Table 1 in addition to world conservation status according to International Union for Conservation of Nature (IUCN) and names of birds in Persian language are presented.

## Discussion

This study is the largest epidemiological study to date performed in Iran. However, low number of collected lice from birds could be due to the fact that most of the ectoparasites including lice leave dead hosts rather quickly. Data reported herein contribute to our knowledge about diversity of avian chewing lice from wild birds in Iran and in a broader context in western Asia. Lice species in this study belonged to both Ischnocera (15 species), Amblycera (14 species). We compiled our data and previous information about avian lice species in Iran in Table 2. So far, lice infestation of birds belonging to 16 orders, 33 families, 60 genera, and 78 species and subspecies has been recorded from Iran. In Supplementary Table 2, the information in Table 2 in addition to world conservation status and names of birds in Persian language are presented.

**Table 2:** Louse species reported from Iran according to their avian hosts until December 2023.

Avian host scientific name	Avian host vernacular name	Lice species	Reference
**ACCIPITRIFORMES**
**Accipitridae**
*Aquila chrysaetos* (Linnaeus, 1758)	Golden eagle	*Craspedorrhynchus aquilinus* (Denny, 1842)	This study (37, 38)
		*Laemobothrion maximum* (Scopoli, 1763)	
		*Laemobothrion* sp.	
*Aquila fasciata* (Vieillot, 1822)	Bonelli’s eagle	*Laemobothrion maximum* (Scopoli, 1763)	Reported the bird as *Hieraaetus fasciatus* (39)
*Aquila heliaca* (Savigny, 1809)	Asian imperial eagle	*Laemobothrion maximum* (Scopoli, 1763)	This study
*Aquila nipalensis* (Hodgson, 1833)	Steppe eagle	*Colpocephalum impressum* (Rudow, 1866)	This study (40)
		*Falcolipeurus suturalis* (Rudow, 1869)	
		*Laemobothrion maximum* (Scopoli, 1763)	
		*Craspedorrhynchus* sp.	
*Aquila rapax* (Temminck, 1828)	Tawny eagle	*Laemobothrion vulturis* (Fabricius, 1775)	This study
		*Colpocephalum impressum* (Rudow, 1866)	
		*Nosopon chanabense* (Ansari, 1951)	
*Buteo buteo* (Linnaeus, 1758)	Buzzard	*Degeeriella fulva* (Giebel, 1874)	This study
		*Degeeriella fusca* (Denny, 1842)	
		*Cuclotogaster heterographus* (Nitzsch, 1866)	
		*Craspedorrhynchus platystomus* (Burmeister, 1838)	
		*Colpocephalum nanum* (Piaget, 1890)	
		*Colpocephalum turbinatum* (Denny, 1842)	
		*Laemobothrion maximum* (Scopoli, 1763)	
*Buteo rufinus* (Cretzschmar, 1829)	Long-legged buzzard	*Trinoton* sp.^×^	This study (17)
		*Laemobothrion maximum* (*Scopoli*, *1763*)	
*Circus aeruginosus* (Linnaeus, 1758)	Eurasian marsh-harrier	*Nosopon lucidum* (Rudow, 1869)	This study
*Gyps fulvus* (Hablizl, 1783)	Eurasian griffon vulture	*Trinoton* sp.^×^	This study (16)
		*Laemobothrion vulturis* (Fabricius, 1775)	
		*Colpocephalum gypsi* (Eichler & Zlotorzycka, 1971)	
		*Colpocephalum* spp.	
		*Falcolipeurus quadripustulatus* (Burmeister, 1838)	
		*Aegypoecus trigonoceps* (Giebel, 1874)	
*Neophron percnopterus* (Linnaeus, 1758)	Egyptian vulture	*Laemobothrion vulturis* (Fabricius, 1775)	(15)
**ANSERIFORMES**
**Anatidae**
*Anas clypeata* (Linnaeus, 1758)	Northern shoveler	*Anaticola crassicornis* (Scopoli, 1763)	This study (38)
		*Pectinopygus* spp.	
*Anas crecca* (Linnaeus, 1758)	Common Teal	*Trinoton querquedulae* (Linnaeus, 1758)	This study
		*Anaticola crassicornis* (Scopoli, 1763)	
*Anas platyrhynchos* (Linnaeus, 1758)	Mallard	*Lipeurus squalidus* (Piaget, 1880)^×^	This study (41, 42)
		*Menacanthus stramineus* (Nitzsch, 1818)^×^	
		*Trinoton anserinum* (Fabricius, 1805)^×^	
		*Anatolica crassicornis* (*Scopoli*, 1763)	
*Anas penelope* Linnaeus, 1758	Eurasian wigeon	*Laemobothrion* spp.	This study
*Anas querquedula* (Linnaeus, 1758)	Garganey	*Trinoton querquedualea* (Linnaeus, 1758)	This study
*Anser anser* (Linnaeus, 1758)	Greylag goose	*Anaticola anseris* (Linnaeus, 1758)	(15, 41, 43)
		*Trinoton anserinum* (Fabricius, 1805)	
		*Cuclotogaster heterographus* (Nitzsch, 1866) ^×^. Also reported as *Liperus heterographus*^×^	
		*Lipeurus caponis* (Linnaeus, 1758)^×^	
		*Menopon gallinae* (Linnaeus, 1758)^×^	
**BUCEROTIFORMES**
**Upupidae**
*Upupa epops* (Linnaeus, 1758)	Hoopoe	*Upupicola upupae* (Schrank, 1803)	(38)
**CHARADRIIFORMES**
**Laridae**
*Chroicocephalus ridibundus* (Linnaeus, 1766)	Black-headed gull	*Austromenopon transversum* (Denny, 1842)	Reported the bird as *Larus ridibundus* (13)
*Sterna hirundo* (Linnaeus, 1758)	Tern	*Quadraceps legatus* (Timmermann, 1952)	This study (12)
		*Saemundssonia meridiana* (Timmermann, 1950)	This study
*Tringa stagnatilis* (Bechstein, 1803)	Marsh sandpiper	*Quadraceps obscurus* (Burm, 1838)	This study
**Recurvirostridae**
*Himantopus himantopus* (Linnaeus, 1758)	Black-winged stilt	*Actornithophilus uniseriatus* (Piaget, 1880) *Quadraceps* spp.	This study
**Scolopacidae**
*Philomachus pugnax* (Linnaeus, 1758)	Ruff	*Lunaceps holophaeus* Burmeister, 1838	This study
		*Actornithophilus cornutus* (Giebel, 1866)	This study
**COLUMBIFORMES**
**Columbidae**
*Columba livia* subsp. *domestica* (Gmelin, 1789)	Domestic pigeon	*Campanulotes compar* (Burmeister, 1838). Also reported as *Goniocotes bidentatus*	(41, 44–46)
		*Columbicola columbae* (Linnaeus, 1758)	
		*Columbicola tschulyschman* (Eichler, 1942)	
		*Lipeurus caponis* (Linnaeus, 1758)^×^	
		*Menopon gallinae* (Linnaeus, 1758)^×^	
		*Menacanthus stramineus* (Nitzsch, 1818)^×^. Also reported as *Menopon stramineum*^×^	
*Columba livia* subsp. *livia* (Gmelin, 1789)	Rock dove	*Campanulotes compar* (Burmeister, 1838)	(13, 47)
		*Colpocephalum turbinatum* Denny, 1842	
		*Columbicola columbae* (Linnaeus, 1758)	
		*Hohorstiella lata* (Piaget, 1880)	
		*Menacanthus stramineus* (Nitzsch, 1818)^×^	
		*Menopon gallinae* (Linnaeus, 1758)^×^	
		*Goniodes* sp.	
*Streptopelia senegalensis* (Linnaeus, 1766)	Laughing dove	*Columbicola columbae* (Linnaeus, 1758)	(48)
*Streptopelia turtur* (Linnaeus, 1758)	European turtle dove	*Colpocephalum pectinatum* (Osborn, 1902)	(38)
		*Strigiphilus* sp.^×^	
**CORACIIFORMES**
**Alcedinidae**
*Alcedo atthis* (Linnaeus, 1758)	Kingfisher	*Alcedoecus annulatus* (Ansari, 1955)	(38)
**Meropidae**
*Merops apiaster* (Linnaeus, 1758)	Bee-eater	*Meromenopon meropis* Clay & Meinertzhagen, 1941	(49)
		*Meropoecus meropis* (Denny, 1842)	
		*Meropsilla apiastri* (Denny, 1842). Reported as *Brueelia apiastri*	
*Merops persicus* (Pallas, 1773)	Blue-cheeked bee-eater	*Meromenopon meropis* Clay & Meinertzhagen, 1941	(49)
		*Meropoecus meropis* (Denny, 1842)	
		*Meropsiella erythropteri* (Piaget, 1885). Reported as *Brueelia erythropteri*	
**CUCULIFORMES**
**Cuculidae**
*Cuculus canorus* (Linnaeus, 1758)	Cuckoo	*Cuculoecus latifrons* (Denny, 1842). Reported as *Philopterus latifron*	(11)
**FALCONIFORMES**
**Falconidae**
*Falco cherrug* (Gray, JE, 1834)	Saker falcon	*Colpocephalum* sp.	(38)
*Falco tinnunculus* (Linnaeus, 1758)	Kestrel	*Laemobothrion maximum* (Scopoli, 1763)^×^	This study (38)
**GALLIFORMES**
**Phasianidae**
*Coturnix coturnix* (Linnaeus, 1758)	Quail	*Amyrsidea fulvomaculata* (Denny, 1842)	(38)
*Gallus gallus domesticus* (Linnaeus, 1758)	Chicken	*Cuclotogaster heterographus* (Nitzsch, 1866). Also reported as *Lipeurus heterographus*	(18, 41, 46, 50–59)
		*Goniodes dissimilis* Denny, 1842	
		*Goniocotes gallinae* (de Geer, 1778)	
		*Goniodes gigas* (Taschenberg, 1879). Also reported as *Goniocotes gigas*	
		*Lipeurus caponis* (Linnaeus, 1758)	
		*Menacanthus pallidulus* (Neumann, 1912). Also reported as *Menopon pallidulum*	
		*Menacanthus stramineus* (Nitzsch, 1818). Also reported as *Menopon stramineum*	
		*Menopon gallinae* (Linnaeus, 1758)	
		*Goniodes* sp.	
		*Lipeurus* sp.	
		*Menopon* sp.	
*Meleagris gallopavo* (Linnaeus, 1758)	Turkey	*Chelopistes meleagridis* (Linnaeus, 1758)	(41, 46, 60)
		*Goniocotes gallinae* (de Geer, 1778)	
		*Goniodes gigas* (Taschenberg, 1879)	
		*Menacanthus stramineus* (Nitzsch, 1818)	
		*Menopon gallinae* (Linnaeus, 1758)	
*Phasianus colchicus* (Linnaeus, 1758)	Pheasant	*Amyrsidea perdicis* (Denny, 1842). Reported as *Amyrsidea hexapilosus*	(38)
*Pavo cristatus* (Linnaeus, 1758)	Peafowl	*Goniodes pavonis* (Linnaeus, 1758)	(61)
*Perdix perdix* (Linnaeus, 1758)	Grey partridge	*Lipeurus* sp.	(62)
		*Menacanthus* sp.	
		*Menopon* sp.	
**GRUIFORMES**
**Rallidae**
*Rallus aquaticus* (Linnaeus, 1758)	Water rail	*Rallicola cuspidatus* (Scopoli, 1763)	This study
*Fulica atra* Linnaeus, 1758	Coot	*Laemobothrion atrum* (Nitzsch, 1818)	This study
**PASSERIFORMES**
**Acrocephalidae**
*Acrocephalus stentoreus* (Hemprich & Ehrenberg, 1833)	Clamorous reed warbler	*Brueelia* sp.	(14)
**Alaudidae**
*Calandrella rufescens* (Vieillot, 1819)	Lesser short-toed lark	*Menacanthus* sp.	(14)
*Galerida cristata* (Linnaeus, 1758)	Crested lark	*Brueelia* sp.	(14)
		*Ricinus* sp.	
**Corvidae**
*Corvus corax* (Linnaeus, 1758)	Raven	*Myrsidea anaspila* (Nitzsch, 1866)	(11, 15)
		*Philopterus corvi* (Linnaeus, 1758)	
		*Cuclotogaster heterographus* (Nitzsch, 1866)^×^	
*Corvus corone* (Linnaeus, 1758)	Carrion crow	*Philopterus ocellatus* (Scopoli, 1763)	(11, 14, 63)
		*Brueelia* sp.	
		*Cuculoecus latifrons* (Denny, 1842). Also reported as *Philopterus latifron*^×^	
*Pica pica* (Linnaeus, 1758)	Black-billed magpie	*Philopterus picae* (Denny, 1842)	(38)
**Emberizidae**
*Emberiza bruniceps* (Brandt, 1841)	Red-headed bunting	*Sturnidoecus rostratus* (Mey, 1982)	(14)
		*Menacanthus* sp.	
*Emberiza calandra* (Linnaeus, 1758)	Corn bunting	*Sturnidoecus rostratus* (Mey, 1982)	(14)
		*Brueelia* sp.	
**Fringillidae**
*Chloris chloris* (Linnaeus, 1758)	European greenfinch	*Myrsidea* sp.	(14)
*Rhodospiza obsolete* (Lichtenstein, 1823)	Desert finch	*Brueelia gobiensis* Mey, 1982	Reported the bird as *Carduelis obsoleta* (14)
		*Philopterus* sp.	
*Fringilla coelebs* (Linnaeus, 1758)	Chaffinch	*Philopterus fringillae* (Scopoli, 1772)	(14)
		*Brueelia* sp.	
**Muscicapidae**
*Saxicola torquatus* (Linnaeus, 1766)	African stonechat	*Brueelia* sp.	(14)
*Luscinia megarhynchos* (Brehm, 1831)	Nightingale	*Brueelia* sp.	(14)
*Oenanthe lugens* (Lichtenstein, 1823)	Mourning wheatear	*Philopterus* sp.	(14)
**Paridae**
*Parus major* (Linnaeus, 1758)	Great tit	*Philopterus pallescens* (Denny, 1842)	(14)
**Passeridae**
*Gymnoris xanthocollis* (Burton, 1838)	Yellow-throated sparrow	*Philopterus fringillae* (Scopoli, 1772)	Reported the bird as *Petronia xanthocollis* (14)
*Passer domesticus* (Linnaeus, 1758)	House sparrow	*Brueelia cyclothorax* (Burmeister, 1838). Reported as *Brueelia subtilis* (Nitzsch, 1874)	(14)
		*Philopterus fringillae* (Scopoli, 1772)	
		*Sturnidoecus refractariolus* (Zlotorzycka, 1964)	
*Passer montanus* (Linnaeus, 1758)	Eurasian sparrow	*Brueelia cyclothorax* (Burmeister, 1838). Reported as *Brueelia subtilis* (Nitzsch, 1874)	(14)
		*Philopterus montani* (Zlotorzycka, 1964)	
		*Sturnidoecus ruficeps* (Nitzsch, 1866)	
		*Campanulotes compar* (Burmeister, 1838)^×^	
*Petronia petronia* (Linnaeus, 1766)	Rock petronia	*Sturnidoecus refractariolus* (Zlotorzycka, 1964)	(14)
		*Brueelia* sp.	
		*Philopterus* sp.	
**Phylloscopidae**
*Phylloscopus collybita* (Vieillot, 1817)	Chiffchaff	*Brueelia* sp.	(14)
		*Menacanthus* sp.	
		*Philopterus* sp.	
		*Sturnidoecus* sp.	
*Phylloscopus nitidus* (Blyth, 1843)	Green warbler	*Brueelia* sp.	(14)
		*Menacanthus* sp.	
**Sturnidae**
*Acridotheres tristis* (Linnaeus, 1766)	Myna	*Brueelia chayanh* Ansari, 1955	(14, 40)
		*Myrsidea invadens* (Kellogg & Chapman, 1902)	
*Sturnus vulgaris* (Linnaeus, 1758)	Starling	*Brueelia nebulosa* (Burmeister, 1838)	(14)
**Sylviidae**
*Sylvia communis* (Latham, 1787)	Whitethroat	*Sturnidoecus* sp.	(14)
**Turdidae**
*Turdus ruficollis* (Pallas, 1776)	Black-throated Thrush	*Philopterus* sp.	(38)
*Turdus merula* (Linnaeus, 1758)	Blackbird	*Ricinus* sp.	(14)
**PELECANIFORMES**
**Ardeidae**
*Ardea purpurea* (Linnaeus, 1766)	Purple heron	*Menacanthus* sp.^×^	(13)
*Egretta garzetta* (Linnaeus, 1766)	Little egret		
		*Ardeicola* sp. Probably *Ardeicola expallidus* Blagoveshtchensky, 1940	(13)
		*Ciconiphilus decimfasciatus* (Boisduval & Lacordaire, 1835)	
**Pelecanidae**
*Pelecanus onocrotalus* (Linnaeus, 1758)	Great white pelican	*Piagetiella titan* (Piaget, 1880)	(4), this study
*Pelecanus crispus* (Bruch, 1832)	Dalmatian pelican	*Colpocephalum eucarenum* (*Burmeister*, *1838*)	
**PHOENICOPTERIFORMES**
**Phoenicopteridae**
*Phoenicopterus ruber* (Linnaeus, 1758)	American flamingo	*Colpocephalum heterosoma*, (Clay, 1951)	This study
**PODICIPEDIFORMES**
**Podicipedidae**
*Podiceps cristatus* (Linnaeus, 1758)	Great crested grebe	*Aquanirmus podicipis* (Denny, 1842)	(13)
		*Pseudomenopon dolium* (Rudow, 1869)	
**Scolopacidae**
*Numenius arquata* (Linnaeus, 1758)	Curlew	*Cummingsiella ovalis* (Scopoli, 1763)	(11, 12, 15)
		*Quadraceps obtusus* (Kellogg & Kuwana, 1902)	
		*Saemundssonia scolopacis phaeopodis* subsp. *humeralis* (Denny, 1842)	
*Scolopax rusticola* (Linnaeus, 1758)	Eurasian woodcock	*Lipeurus* sp.	(19)
		*Philopterus* sp.	
**STRIGIFORMES**
**Strigidae**
*Asio otus* (Linnaeus, 1758)	Long-eared owl	*Strigiphilus* sp.	This study
*Athene noctua* (Scopoli, 1769)	Little owl	*Colpocephalum pectinatum* (Osborn, 1902)	(38)
		*Philopterus ocellatus* (Scopoli, 1763)^×^	
*Bubo bubo* (Linnaeus, 1758)	Eagle owl	*Strigiphilus strigis* (Pontoppidan, 1763)	This study
**SULIFORMES**
**Phalacrocoracidae**
*Phalacrocorax carbo* (Linnaeus, 1758)	Cormorant	*Pectinopygus gyricornis* (Denny, 1842)	(13)

Names of orders are capitalized, and names of families are showed in bold. ^×^The louse species is not normally found on this bird. Its report is probably due to contamination or misidentification.

Review of all relevant publications indicated that in some reports from Iran, researchers identified the lice specimens only to genus level, i.e., *Brueelia* (nine documents), *Philopterus*, *Menacanthus* (six documents), *Ricinus*, *Lipeurus* (three documents), *Sturnidoecus*, *Trinoton*, *Menopon*, and *Goniodes* (two documents), *Ardeicola*, *Colpocephalum*, *Craspedorrhynchus*, *Laemobothrion*, *Strigiphilus*, and *Myrsidea* (one document) (14, 16, 17, 19, 38, 40, 47, 50, 51, 62). The reason could be damage of the specimens, observation of a louse with morphological differences from identification keys or difficulty in identification of the species. It is necessary that researchers will try their best to identify the lice to species level correctly and provide the drawings, measurements, or photos.

Observation of one male poultry head louse specimen, *Cuclotogaster heterographus* (Nitzsch, 1866) which was collected from the buzzard *Buteo buteo* (Linnaeus, 1758) in this study was probably because the buzzard preyed with a galliform bird and the louse was mechanically transferred to the predator. In some reports from Iran, lice species that normally infest other bird orders were documented on abnormal bird species. For instance, *Trinoton* sp. that infest Anseriform birds were collected from raptors *Buteo rufinus* and *Gyps fulvus* (16, 17). It can be assumed that lice infestation occurred during feeding the raptors from their preys. In addition, in some reports, *Menacanthus stramineus*, *Menopon gallinae*, and *Cuclotogaster heterographus* that live on Galliformes were collected from mallards and geese (42, 43) as well as pigeons (47). These findings could be due to keeping mixed species together by nomads which is a normal practice in Iran although misidentification cannot be ruled out. Special caution should be taken for interpretation of such findings.

It is known that both amblyceran and ischnoceran lice can act as vectors or intermediate hosts of helminths, bacteria, and viruses, so it was suggested to delouse the wild birds with insecticides (1) however, we disagree with manipulating host–parasite interactions in the wildlife. Additionally, from the conservation point of view some authors expressed their concerns about co-extinction of the lice with their hosts, e.g., *Rallicola extinctus* (64) and their extinction during the conservation efforts to save the host, e.g., *Rallicola pilgrimi* (Clay, 1972) and *Colpocephalum californici* (31, 64, 65). According to International Union for Conservation of Nature (IUCN), there are concerns regarding decreasing population of several predators such as *Aquila nipalensis* Hodgson, 1833 steppe eagle (endangered) and *Aquila heliaca* Savigny, and 1809 Asian imperial eagle (vulnerable) (66). Hence, it is suggested that conservationists consider preserving host-specific lice as part of their efforts to save vertebrate hosts (65).

This study provides the first information about lice infestation of wild birds in different regions of Iran and reports *Craspedorrhynchus platystomus*, *Colpocephalum nanum*, *Colpocephalum gypsi*, *Colpocephalum eucarenum*, *Colpocephalum heterosoma*, *Degeeriella fulva*, *Degeeriella fusca*, *Nosopon chanabense*, *Nosopon lucidum*, *Falcolipeurus quadripustulatus Falcolipeurus suturalis*, *Aegypoecus trigonoceps*, *Trinoton querquedulae*, *Anaticola crassicornis*, *Quadraceps obscurus*, *Rallicola cuspidatus*, and *Strigiphilus strigis* for the first time from the country. Review of the published data on avian lice fauna of Iran shows that the information is available for almost 14% of the bird species. In contrast, researchers from the neighboring country Turkey have identified over 150 lice species from more than half of the bird species inhabiting the country (21). As Iran and Turkey share many bird species, it seems that many louse species remain to be discovered. Molecular phylogenetic analysis of avian lice from Iran will bring clearer understanding of the role of migratory birds in biogeographic distributions.

## Data availability statement

The original contributions presented in the study are included in the article/Supplementary material, further inquiries can be directed to the corresponding author.

## Ethics statement

Ethical approval was not required for the study involving animals in accordance with the local legislation and institutional requirements because examined birds Hamedan province were euthanized by a certified veterinarian of the Provincial Department of Environment because of general health failure prior to transfer to the Faculty of Veterinary Medicine, Bu-Ali Sina University. Birds in other provinces were dead animals.

## Author contributions

ZB: Methodology, Writing – original draft. AS: Conceptualization, Funding acquisition, Investigation, Methodology, Project administration, Resources, Supervision, Validation, Writing – original draft, Writing – review & editing. JK: Methodology, Writing – original draft. MB: Methodology, Writing – original draft. EM: Methodology, Writing – original draft. BD: Investigation, Methodology, Supervision, Writing – original draft, Writing – review & editing.

## Footnotes

^1^ http://phthiraptera.myspecies.info/

^2^ www.pubmed.gov

^3^ www.google.com

^4^ www.sid.ir

^5^ https://irandoc.ac.ir/

^6^ https://www.civilica.com/
