# Supplementary material for: Chewing lice of wild birds in Iran: new data and a checklist of avian louse species reported in Iran
Source: Front Vet Sci. 2024 Feb 2;10:1324619. doi: 10.3389/fvets.2023.1324619 (PMC10869535; doi:10.3389/fvets.2023.1324619)
Supplement: Supplementary file 1 [file Table_1.docx]

**Supplementary Table 1.** Table 1 supplemented with conservation status and names of birds in Persian language. Distribution of louse species of wild birds in some regions of Iran (September 2019 and December 2023) according to their host bird species. Names of orders are capitalized, and names of families are typed in bold.

| ***n* birds examined** | **Host bird taxonomy** | **Host scientific name** | **Host vernacular name** | **Global conservation status^a^** | **نام فارسی** | **Louse species** | **Suborder** | **Family** | **City** | ***n* total/infested** | **Louse prevalence (*n*)** | | | | |
| --- | --- | --- | --- | --- | --- | --- | --- | --- | --- | --- | --- | --- | --- | --- | --- |
|  |  |  |  |  |  |  |  |  |  |  | **Male** | **Female** | **Nymph** | **Damaged** | **Total** |
|  | ACCIPITRIFORMES |  |  |  | بازسانان |  |  |  |  |  |  |  |  |  |  |
| 1 | **Accipitridae** | *Accipiter* *badius* (Gmelin, 1788) | Shikra | Least concern | پیغوی کوچک | - | - | - | Zahedan | 0/1 |  |  |  |  |  |
|  |  |  |  |  |  |  |  |  |  |  |  |  |  |  |  |
| 8 |  | *Accipiter nisus* (Linnaeus, 1758) | Eurasian sparrowhawk | Least concern | قرقی | - | - | - | Hamun | 0/2 |  |  |  |  |  |
|  |  |  |  |  |  |  |  |  | Hamedan | 0/5 |  |  |  |  |  |
|  |  |  |  |  |  |  |  |  | Zabol | 0/1 |  |  |  |  |  |
|  |  |  |  |  |  |  |  |  |  |  |  |  |  |  |  |
| 1 |  | *Aegypius* *monachus* (Linnaeus, 1766) | Black vulture | Near Threatened | دال سیاه | - | - | - | Zahedan | 0/1 |  |  |  |  |  |
|  |  |  |  |  |  |  |  |  |  |  |  |  |  |  |  |
| 4 |  | *Aquila chrysaetos* (Linnaeus, 1758) | Golden eagle | Least concern | عقاب طلایی | *Craspedorrhynchus aquilinus* (Denny, 1842) | Ischnocera | Philopteridae | Hamedan | 3/3 | 5 | 4 | 0 | 0 | 9 |
|  |  |  |  |  |  |  |  |  | Kerman | 0/1 |  |  |  |  |  |
|  |  |  |  |  |  |  |  |  |  |  |  |  |  |  |  |
| 1 |  | *Aquila heliaca* Savigny, 1809 | Asian imperial eagle | Vulnerable | عقاب شاهی | *Laemobothrion maximum* (Scopoli, 1763) | Amblycera | Laemobothriidae | Hamedan | 1/1 | 0 | 0 | 3 | 0 | 3 |
|  |  |  |  |  |  |  |  |  |  |  |  |  |  |  |  |
| 3 |  | *Aquila nipalensis* Hodgson, 1833 | Steppe eagle | Endangered | عقاب صحرایی | *Laemobothrion maximum* (Scopoli, 1763) | Amblycera | Laemobothriidae | Hamedan | 2/3 | 2 | 4 | 0 | 0 | 6 |
|  |  |  |  |  |  | *Laemobothrion vulturis* (Fabricius, 1775) |  |  |  | 1/3 | 0 | 2 | 2 | 0 | 4 |
|  |  |  |  |  |  | *Colpocephalum impressum* Rudow, 1866 |  | Menoponidae |  | 1/3 | 0 | 1 | 0 | 0 | 1 |
|  |  |  |  |  |  | *Craspedorrhynchus aquilinus* (Denny, 1842) | Ischnocera | Philopteridae |  | 1/3 | 0 | 1 | 0 | 0 | 1 |
|  |  |  |  |  |  | *Falcolipeurus suturalis* (Rudow, 1869) |  |  |  | 1/3 | 0 | 1 | 0 | 0 | 1 |
|  |  |  |  |  |  |  |  |  |  |  |  |  |  |  |  |
| 1 |  | *Aquila rapax* (Temminck, 1828) | Tawny eagle | Least concern | عقاب خاکی (دشتی) | *Laemobothrion vulturis* (Fabricius, 1775) | Amblycera | Laemobothriidae | Hamedan | 1/1 | 1 | 0 | 0 | 0 | 1 |
|  |  |  |  |  |  | *Colpocephalum impressum* Rudow, 1866 |  | Menoponidae |  | 1/1 | 4 | 0 | 1 | 0 | 5 |
|  |  |  |  |  |  | *Nosopon chanabense* (Ansari, 1951) |  |  |  | 1/1 | 0 | 2 | 0 | 0 | 2 |
|  |  |  |  |  |  |  |  |  |  |  |  |  |  |  |  |
| 14 |  | *Buteo buteo* (Linnaeus, 1758) | Buzzard | Least concern | سارگپه معمولی | *Degeeriella fulva* (Giebel, 1874) | Ischnocera | Philopteridae | Hamedan | 1/12 | 10 | 8 | 0 | 0 | 18 |
|  |  |  |  |  |  | *Degeeriella fusca* (Denny, 1842) |  |  |  | 1/12 | 2 | 4 | 1 | 0 | 7 |
|  |  |  |  |  |  | *Cuclotogaster heterographus* (Nitzsch, 1866) |  |  |  | 1/12 | 1 | 0 | 0 | 0 | 1 |
|  |  |  |  |  |  | *Craspedorrhynchus platystomus* (Burmeister, 1838) |  |  |  | 2/12 | 18 | 19 | 3 | 0 | 40 |
|  |  |  |  |  |  | *Colpocephalum nanum* Piaget, 1890 | Amblycera | Menoponidae |  | 1/12 | 0 | 2 | 2 | 1 | 5 |
|  |  |  |  |  |  | *Colpocephalum turbinatum* Denny, 1842 |  |  |  | 1/12 | 24 | 12 | 0 | 0 | 36 |
|  |  |  |  |  |  | *Laemobothrion maximum* (Scopoli, 1763) |  | Laemobothriidae |  | 1/12 | 3 | 3 | 6 | 0 | 12 |
|  |  |  |  |  |  |  |  |  | Kerman | 0/2 |  |  |  |  |  |
|  |  |  |  |  |  |  |  |  |  |  |  |  |  |  |  |
| 3 |  | *Buteo rufinus* (Cretzschmar, 1829) | The long-legged buzzard | Least concern | سارگپه پا بلند | *Laemobothrion maximum* (Scopoli, 1763) | Amblycera | Laemobothriidae | Kerman | 1/1 | 1 | 1 | 1 | 0 | 3 |
|  |  |  |  |  |  |  |  |  | Zabol | 0/1 |  |  |  |  |  |
|  |  |  |  |  |  |  |  |  | Zahedan | 0/1 |  |  |  |  |  |
|  |  |  |  |  |  |  |  |  |  |  |  |  |  |  |  |
| 3 |  | *Circus aeruginosus* (Linnaeus, 1758) | Eurasian marsh-harrier | Least concern | سنقر تالابی | *Nosopon lucidum* (Rudow, 1869) | Amblycera | Menoponidae | Hamedan | 1/3 | 6 | 12 | 2 | 0 | 20 |
|  |  |  |  |  |  |  |  |  |  |  |  |  |  |  |  |
| 2 |  | *Circaetus gallicus* (Gmelin, 1788) | Short-toed snake eagle | Least concern | عقاب مارخور پنجه‌کوتاه | - | - | - | Zahedan | 0/2 |  |  |  |  |  |
|  |  |  |  |  |  |  |  |  |  |  |  |  |  |  |  |
| 4 |  | *Gyps fulvus* (Hablizl, 1783) | Griffon vulture | Least concern | دال | *Laemobothrion vulturis* (Fabricius, 1775) | Amblycera | Laemobothriidae | Hamedan | 2/4 | 1 | 5 | 4 | 0 | 10 |
|  |  |  |  |  |  | *Colpocephalum gypsi* (Eichler & Zlotorzycka, 1971) |  | Menoponidae | Zabol | 1/4 | 1 | 0 | 0 | 0 | 1 |
|  |  |  |  |  |  | *Colpocephalum* spp. |  |  |  | 1/4 | 0 | 0 | 0 | 1 | 1 |
|  |  |  |  |  |  | *Falcolipeurus quadripustulatus* (Burmeister, 1838) | Ischnocera | Philopteridae |  | 2/4 | 23 | 18 | 0 | 0 | 41 |
|  |  |  |  |  |  | *Aegypoecus trigonoceps* (Giebel, 1874) |  |  | Kerman | 1/4 | 3 | 3 | 1 | 0 | 7 |
|  | ANSERIFORMES |  |  |  | غازسانان |  |  |  |  |  |  |  |  |  |  |
| 6 | **Anatidae** | *Aythya ferina* (Linnaeus, 1758) | Common pochard | Vulnerable | اردک سرحنایی (یا) بلغی | - | - | - | Zehak | 0/3 |  |  |  |  |  |
|  |  |  |  |  |  |  |  |  | Zabol | 0/1 |  |  |  |  |  |
|  |  |  |  |  |  |  |  |  | Chabahar | 0/2 |  |  |  |  |  |
|  |  |  |  |  |  |  |  |  |  |  |  |  |  |  |  |
| 10 |  | *Anas crecca* Linnaeus, 1758 | Common teal | Least concern | خوتکا | *Trinoton querquedulae* (Linnaeus, 1758) | Amblycera | Menoponidae | Zehak | 5/8 | 0 | 3 | 0 | 0 | 3 |
|  |  |  |  |  |  | *Anaticola crassicornis* (Scopoli, 1763) | Ischnocera | Philopteridae | Chabahar | 0/2 | 3 | 5 | 1 | 0 | 9 |
|  |  |  |  |  |  |  |  |  |  |  |  |  |  |  |  |
| 7 |  | *Anas platyrhynchos* Linnaeus, 1758 | Mallard | Least concern | اردک گردن سبز (یا) سوزگردن | *Anaticola crassicornis* (Scopoli, 1763) | Ischnocera | Philopteridae | Zehak | 0/1 | 3 | 1 | 0 | 0 | 4 |
|  |  |  |  |  |  |  |  |  | Zabol | 2/2 |  |  |  |  |  |
|  |  |  |  |  |  | *Trinoton querquedulae* (Linnaeus, 1758) | Amblycera | Menoponidae | Hamedan | 1/1 | 0 | 1 | 0 | 0 | 1 |
|  |  |  |  |  |  |  |  |  | Chabahar | 0/3 |  |  |  |  |  |
|  |  |  |  |  |  |  |  |  |  |  |  |  |  |  |  |
| 1 |  | *Anas* *penelope* Linnaeus, 1758 | Eurasian wigeon | Least concern |  | *Laemobothrion* spp. | Amblycera | Laemobothriidae | Zabol | 1/1 | 0 | 0 | 1 | 0 | 1 |
|  |  |  |  |  |  |  |  |  |  |  |  |  |  |  |  |
| 9 |  | *Spatula clypeata* (Linnaeus, 1758) | Northern shoveler | Least concern | کمچه (یا) اردک نوک پهن | *Pectinopygus* spp. | Ischnocera | Philopteridae | Zabol | 1/3 | 0 | 1 | 0 | 0 | 1 |
|  |  |  |  |  |  |  |  |  | Zehak | 0/3 |  |  |  |  |  |
|  |  |  |  |  |  |  |  |  | Chabahar | 0/3 |  |  |  |  |  |
|  |  |  |  |  |  |  |  |  |  |  |  |  |  |  |  |
| 1 |  | *Mergus* *merganser* (Linnaeus, 1758) | Common merganser | Least concern | اردک ماهی‌خوار | - | - | - | Zabol | 0/1 |  |  |  |  |  |
|  |  |  |  |  |  |  |  |  |  |  |  |  |  |  |  |
| 2 |  | *Spatula querquedula* (Linnaeus, 1758) | Garganey | Least concern | خوتکا ابرو سفید | *Trinoton querquedulae* (Linnaeus, 1758) | Amblycera | Menoponidae | Zehak | 1/2 | 1 | 0 | 0 | 0 | 1 |
|  |  |  |  |  |  |  |  |  |  |  |  |  |  |  |  |
| 2 |  | *Tadorna tadorna* (Linnaeus, 1758) | Common shelduck | Least concern | تنجه (یا) عروس مرغابی | - | - | - | Zehak | 0/2 |  |  |  |  |  |
|  | BUCEROTIFORMES |  |  |  | نوک‌شاخ‌سانان |  |  |  |  |  |  |  |  |  |  |
| 3 | **Upupidae** | *Upupa epops* Linnaeus, 1758 | Eurasian hoopoe | Least concern | هد هد (یا) شانه به سر | - | - | - | Nehbandan | 0/1 |  |  |  |  |  |
|  |  |  |  |  |  |  |  |  | Birjand | 0/1 |  |  |  |  |  |
|  |  |  |  |  |  |  |  |  | Zabol | 0/1 |  |  |  |  |  |
|  | CHARADRIIFORMES |  |  |  | سلیم­سانان |  |  |  |  |  |  |  |  |  |  |
| 4 | **Recurvirostridae** | *Himantopus* *himantopus* (Linnaeus, 1758) | Black-winged stilt | Least concern | چوب پا | *Actornithophilus* *uniseriatus* (Piaget, 1880) | Amblycera | Menoponidae | Zehak | 4/4 | 1 | 0 | 1 | 0 | 2 |
|  |  |  |  |  |  | *Quadraceps* spp. | Ischnocera | Philopteridae |  |  | 3 | 0 | 1 | 0 | 4 |
|  |  |  |  |  |  |  |  |  |  |  |  |  |  |  |  |
| 3 | **Scolopacidae** | *Philomachus* *pugnax* (Linnaeus, 1758) | Ruff | Least concern | تلیله شکیل | *Lunaceps* *holophaeus* Burmeister, 1838 | Ischnocera | Philopteridae | Zehak | 3/3 | 2 | 2 | 0 | 0 | 4 |
|  |  |  |  |  |  | *Actornithophilus* *cornutus* (Giebel, 1866) | Amblycera | Menoponidae |  |  | 1 | 0 | 0 | 0 | 1 |
|  |  |  |  |  |  |  |  |  |  |  |  |  |  |  |  |
| 2 |  | *Tringa stagnatilis* (Bechstein, 1803) | Marsh sandpiper | Least concern | آبچلیک تالابی | *Quadraceps obscurus* (Burmeister, 1838) | Ischnocera | Philopteridae | Zehak | 2/2 | 3 | 3 | 0 | 0 | 6 |
|  |  |  |  |  |  |  |  |  |  |  |  |  |  |  |  |
| 14 |  | *Phalaropus lobatus* (Linnaeus, 1758) | Red-necked phalarope | Least concern | شناگر گردن‌سرخ | *-* | - | - | Zehak | 0/14 |  |  |  |  |  |
|  |  |  |  |  |  |  |  |  |  |  |  |  |  |  |  |
| 17 | **Laridae** | *Sterna repressa* Hartert, 1916 | White-cheeked tern | Least concern | پرستو دریایی گونه سفید | - | - | - | Zehak | 0/7 |  |  |  |  |  |
|  |  |  |  |  |  |  |  |  | Zabol | 0/4 |  |  |  |  |  |
|  |  |  |  |  |  |  |  |  | Hamun | 0/6 |  |  |  |  |  |
|  | CAPRIMULGIFORMES |  |  |  | شبگردسانان |  |  |  |  |  |  |  |  |  |  |
| 8 | **Caprimulgidae** | *Caprimulgus aegyptius* Lichtenstein, 1823 | Egyptian nightjar | Least concern | شبگرد دشتی | - | - | - | Hamun | 0/3 |  |  |  |  |  |
|  |  |  |  |  |  |  |  |  | Zabol | 0/1 |  |  |  |  |  |
|  |  |  |  |  |  |  |  |  | Nimruz | 0/2 |  |  |  |  |  |
|  |  |  |  |  |  |  |  |  | Zehak | 0/2 |  |  |  |  |  |
|  | COLUMBIFORMES |  |  |  | کبوترسانان |  |  |  |  |  |  |  |  |  |  |
| 22 | **Columbidae** | *Streptopelia decaocto* (Frivaldszky, 1838) | Eurasian collared dove | Least concern | یاکریم اوراسیایی | - | - | - | Hirmand | 0/4 |  |  |  |  |  |
|  |  |  |  |  |  |  |  |  | Zabol | 0/4 |  |  |  |  |  |
|  |  |  |  |  |  |  |  |  | Nimruz | 0/3 |  |  |  |  |  |
|  |  |  |  |  |  |  |  |  | Zehak | 0/6 |  |  |  |  |  |
|  |  |  |  |  |  |  |  |  | Hamun | 0/5 |  |  |  |  |  |
|  |  |  |  |  |  |  |  |  |  |  |  |  |  |  |  |
| 36 |  | *Spilopelia senegalensis* (Linnaeus, 1766) | Laughing dove | Least concern | قمری خانگی | - | - | - | Hirmand | 0/1 |  |  |  |  |  |
|  |  |  |  |  |  |  |  |  | Zabol | 0/8 |  |  |  |  |  |
|  |  |  |  |  |  |  |  |  | Nimruz | 0/2 |  |  |  |  |  |
|  |  |  |  |  |  |  |  |  | Zehak | 0/5 |  |  |  |  |  |
|  |  |  |  |  |  |  |  |  | Sari | 0/1 |  |  |  |  |  |
|  |  |  |  |  |  |  |  |  | Kerman | 0/2 |  |  |  |  |  |
|  |  |  |  |  |  |  |  |  | Zahedan | 0/2 |  |  |  |  |  |
|  |  |  |  |  |  |  |  |  | Mashhad | 0/3 |  |  |  |  |  |
|  |  |  |  |  |  |  |  |  | Kalaleh | 0/2 |  |  |  |  |  |
|  |  |  |  |  |  |  |  |  | Gorgan | 0/2 |  |  |  |  |  |
|  |  |  |  |  |  |  |  |  | Nehbandan | 0/1 |  |  |  |  |  |
|  |  |  |  |  |  |  |  |  | Birjand | 0/1 |  |  |  |  |  |
|  |  |  |  |  |  |  |  |  | Hamun | 0/6 |  |  |  |  |  |
|  | FALCONIFORMES |  |  |  | شاهین شکلان |  |  |  |  |  |  |  |  |  |  |
| 46 | **Falconidae** | *Falco tinnunculus* Linnaeus, 1758 | Kestrel | Least concern | دلیجه معمولی | *Laemobothrion maximum* (Scopoli, 1763) | Amblycera | Laemobothriidae | Hamedan | 1/12 | 0 | 0 | 1 | 0 | 1 |
|  |  |  |  |  |  |  |  |  | Kerman | 0/26 |  |  |  |  |  |
|  |  |  |  |  |  |  |  |  | Zabol | 0/4 |  |  |  |  |  |
|  |  |  |  |  |  |  |  |  | Zahedan | 0/4 |  |  |  |  |  |
|  |  |  |  |  |  |  |  |  |  |  |  |  |  |  |  |
| 4 |  | *Falco cherrug* Gray, 1834 | Saker falcon | Endangered | بالابان | - | - | - | Kerman | 0/4 |  |  |  |  |  |
|  |  |  |  |  |  |  |  |  |  |  |  |  |  |  |  |
| 1 |  | *Falco naumanni* (Fleischer, 1818) | Lesser kestrel | Least concern | دلیجه کوچک | - | - | - | Zabol | 0/1 |  |  |  |  |  |
|  |  |  |  |  |  |  |  |  |  |  |  |  |  |  |  |
| 2 |  | *Falco peregrinus* subsp. *pelegrinoides* Temminck, 1829 | Barbary falcon | Least concern | شاهین بحری | - | - | - | Kerman | 0/2 |  |  |  |  |  |
|  | GALLIFORMES |  |  |  |  |  |  |  |  |  |  |  |  |  |  |
| 22 | **Phasianidae** | *Ammoperdix griseogularis* (Brandt, 1843) | See-see partridge | Least concern | تیهو | - | - | - | Zahedan | 0/8 |  |  |  |  |  |
|  |  |  |  |  |  |  |  |  | Nehbandan | 0/3 |  |  |  |  |  |
|  |  |  |  |  |  |  |  |  | Iranshahr | 0/1 |  |  |  |  |  |
|  |  |  |  |  |  |  |  |  | Khash | 0/3 |  |  |  |  |  |
|  |  |  |  |  |  |  |  |  | Chabahar | 0/1 |  |  |  |  |  |
|  |  |  |  |  |  |  |  |  | Birjand | 0/1 |  |  |  |  |  |
|  |  |  |  |  |  |  |  |  | Qaen | 0/1 |  |  |  |  |  |
|  |  |  |  |  |  |  |  |  | Torbat-Heidarie | 0/2 |  |  |  |  |  |
|  |  |  |  |  |  |  |  |  | Gonaabaad | 0/1 |  |  |  |  |  |
|  |  |  |  |  |  |  |  |  | Saravan | 0/1 |  |  |  |  |  |
|  |  |  |  |  |  |  |  |  |  |  |  |  |  |  |  |
| 11 |  | *Alectoris chukar* (Gray, 1830) | Chukar | Least concern | کبک معمولی (یا) کبک کوهپایه | - | - | - | Iranshahr | 0/3 |  |  |  |  |  |
|  |  |  |  |  |  |  |  |  | Saravan | 0/4 |  |  |  |  |  |
|  |  |  |  |  |  |  |  |  | Chabahar | 0/1 |  |  |  |  |  |
|  |  |  |  |  |  |  |  |  | Zahedan | 0/3 |  |  |  |  |  |
|  |  |  |  |  |  |  |  |  |  |  |  |  |  |  |  |
| 15 |  | *Coturnix coturnix* (Linnaeus, 1758) | Quail | Least concern | بلدرچین | - | - | - | Kerman | 0/14 |  |  |  |  |  |
|  |  |  |  |  |  |  |  |  | Zabol | 0/1 |  |  |  |  |  |
|  |  |  |  |  |  |  |  |  |  |  |  |  |  |  |  |
| 5 |  | *Francolinus francolinus* (Linnaeus, 1766) | Black francolin | Least concern | دراج | - | - | - | Hirmand | 0/1 |  |  |  |  |  |
|  |  |  |  |  |  |  |  |  | Zehak | 0/1 |  |  |  |  |  |
|  |  |  |  |  |  |  |  |  | Hamun | 0/3 |  |  |  |  |  |
|  | GRUIFORMES |  |  |  | درناشکلان |  |  |  |  |  |  |  |  |  |  |
| 1 | **Rallidae** | *Rallus aquaticus* Linnaeus, 1758 | Water rail | Least concern | یلوه­ی آبی | *Rallicola cuspidatus* (Scopoli, 1763) | Ischnocera | Philopteridae | Hamedan | 1/1 | 1 | 0 | 0 | 0 | 1 |
|  |  |  |  |  |  |  |  |  |  |  |  |  |  |  |  |
| 18 |  | *Fulica atra* Linnaeus, 1758 | Coot | Least concern | چور (یا) چنگر | *Laemobothrion* (*Eulaemobothrion*) *atrum* (Nitzsch, 1818) | Amblycera | Laemobothriidae | Zehak | 0/12 |  |  |  |  |  |
|  |  |  |  |  |  |  |  |  | Zabol | 1/3 | 0 | 1 | 0 | 0 | 1 |
|  |  |  |  |  |  |  |  |  | Hamun | 0/2 |  |  |  |  |  |
|  |  |  |  |  |  |  |  |  | Nimruz | 0/1 |  |  |  |  |  |
|  | OTIDIFORMES |  |  |  | هوبره‌ایان |  |  |  |  |  |  |  |  |  |  |
| 1 | **Otididae** | *Chlamydotis* *macqueenii* (Gray, 1832) | MacQueen's bustard | Vulnerable | هوبره آسیایی | - | - | - | Kerman | 0/1 |  |  |  |  |  |
|  |  |  |  |  |  |  |  |  |  |  |  |  |  |  |  |
|  | PASSERIFORMES |  |  |  | گنجشک­سانان |  |  |  |  |  |  |  |  |  |  |
| 4 | **Acrocephalidae** | *Acrocephalus scirpaceus* (Hermann, 1804) | Eurasian reed warbler | Least concern | سسک تالابی معمولی | *-* | - | - | Zehak | 0/2 |  |  |  |  |  |
|  |  |  |  |  |  |  |  |  | Zabol | 0/2 |  |  |  |  |  |
|  |  |  |  |  |  |  |  |  |  |  |  |  |  |  |  |
| 5 | **Alaudidae** | *Alaemon alaudipes* (Desfontaines, 1789) | Greater hoopoe-lark | Least concern | چکاوک هدهدی |  |  |  | Hirmand | 0/2 |  |  |  |  |  |
|  |  |  |  |  |  |  |  |  | Zabol | 0/2 |  |  |  |  |  |
|  |  |  |  |  |  |  |  |  | Zehak | 0/1 |  |  |  |  |  |
|  |  |  |  |  |  |  |  |  |  |  |  |  |  |  |  |
| 11 |  | *Alauda arvensis* Linnaeus, 1758 | Eurasian skylark | Least concern | چکاوک آسمانی | *-* | - | - | Hamun | 0/2 |  |  |  |  |  |
|  |  |  |  |  |  |  |  |  | Zehak | 0/1 |  |  |  |  |  |
|  |  |  |  |  |  |  |  |  | Hirmand | 0/2 |  |  |  |  |  |
|  |  |  |  |  |  |  |  |  | Zabol | 0/4 |  |  |  |  |  |
|  |  |  |  |  |  |  |  |  | Nimruz | 0/2 |  |  |  |  |  |
|  |  |  |  |  |  |  |  |  |  |  |  |  |  |  |  |
| 35 |  | *Galerida cristata* (Linnaeus, 1758) | Crested lark | Least concern | چکاوک کاکلی | *-* | - | - | Hamun | 0/4 |  |  |  |  |  |
|  |  |  |  |  |  |  |  |  | Zabol | 0/4 |  |  |  |  |  |
|  |  |  |  |  |  |  |  |  | Hirmand | 0/4 |  |  |  |  |  |
|  |  |  |  |  |  |  |  |  | Birjand | 0/2 |  |  |  |  |  |
|  |  |  |  |  |  |  |  |  | Neishaboor | 0/1 |  |  |  |  |  |
|  |  |  |  |  |  |  |  |  | Quchan | 0/1 |  |  |  |  |  |
|  |  |  |  |  |  |  |  |  | Torbat-Heidarie | 0/1 |  |  |  |  |  |
|  |  |  |  |  |  |  |  |  | Gonaabaad | 0/1 |  |  |  |  |  |
|  |  |  |  |  |  |  |  |  | Gorgan | 0/2 |  |  |  |  |  |
|  |  |  |  |  |  |  |  |  | Shirvan | 0/2 |  |  |  |  |  |
|  |  |  |  |  |  |  |  |  | Bojnurd | 0/2 |  |  |  |  |  |
|  |  |  |  |  |  |  |  |  | Sari | 0/2 |  |  |  |  |  |
|  |  |  |  |  |  |  |  |  | Qaen | 0/1 |  |  |  |  |  |
|  |  |  |  |  |  |  |  |  | Nimruz | 0/4 |  |  |  |  |  |
|  |  |  |  |  |  |  |  |  | Zehak | 0/4 |  |  |  |  |  |
|  |  |  |  |  |  |  |  |  |  |  |  |  |  |  |  |
| 4 |  | *Melanocorypha calandra* (Linnaeus, 1766) | Calandra lark | Least concern | چکاوک گندم زار | - | - | - | Mashhad | 0/4 |  |  |  |  |  |
|  |  |  |  |  |  |  |  |  |  |  |  |  |  |  |  |
| 7 | **Cisticolidae** | *Prinia gracilis* (Lichtenstein, 1823) | Graceful prinia | Least concern | سسک شکیل | *-* | - | - | Zehak | 0/3 |  |  |  |  |  |
|  |  |  |  |  |  |  |  |  | Hamun | 0/2 |  |  |  |  |  |
|  |  |  |  |  |  |  |  |  | Zabol | 0/2 |  |  |  |  |  |
|  |  |  |  |  |  |  |  |  |  |  |  |  |  |  |  |
| 5 | **Fringillidae** | *Serinus pusillus* (Pallas, 1811) | Red-fronted serin | Least concern | سهره پیشانی سرخ | *-* | - | - | Kerman | 0/5 |  |  |  |  |  |
|  |  |  |  |  |  |  |  |  |  |  |  |  |  |  |  |
| 5 |  | *Carduelis carduelis* (Linnaeus, 1758) | European goldfinch | Least concern | سهره معمولی | *-* | - | - | Mashhad | 0/1 |  |  |  |  |  |
|  |  |  |  |  |  |  |  |  | Birjand | 0/1 |  |  |  |  |  |
|  |  |  |  |  |  |  |  |  | Gorgan | 0/1 |  |  |  |  |  |
|  |  |  |  |  |  |  |  |  | Kalaleh | 0/1 |  |  |  |  |  |
|  |  |  |  |  |  |  |  |  | Kerman | 0/1 |  |  |  |  |  |
|  |  |  |  |  |  |  |  |  |  |  |  |  |  |  |  |
| 11 |  | *Rhodospiza obsoleta* (Lichtenstein, 1823) | Desert finch | Least concern | سهره خاکی | *-* | - | - | Nehbandan | 0/2 |  |  |  |  |  |
|  |  |  |  |  |  |  |  |  | Qaen | 0/3 |  |  |  |  |  |
|  |  |  |  |  |  |  |  |  | Mashhad | 0/3 |  |  |  |  |  |
|  |  |  |  |  |  |  |  |  | Bojnurd | 0/1 |  |  |  |  |  |
|  |  |  |  |  |  |  |  |  | Birjand | 0/1 |  |  |  |  |  |
|  |  |  |  |  |  |  |  |  | Torbat- Heidarieh | 0/1 |  |  |  |  |  |
|  |  |  |  |  |  |  |  |  |  |  |  |  |  |  |  |
| 7 | **Hirundinidae** | *Hirundo rustica* Linnaeus, 1758 | Barn swallow | Least concern | پرستوی معمولی |  |  |  | Hirmand | 0/2 |  |  |  |  |  |
|  |  |  |  |  |  |  |  |  | Hamun | 0/1 |  |  |  |  |  |
|  |  |  |  |  |  |  |  |  | Zehak | 0/4 |  |  |  |  |  |
|  |  |  |  |  |  |  |  |  |  |  |  |  |  |  |  |
| 19 | **Passeridae** | *Passer hispaniolensis* (Temminck, 1820) | Spanish sparrow | Least concern | گنجشک سینه سیاه | *-* | - | - | Nimruz | 0/3 |  |  |  |  |  |
|  |  |  |  |  |  |  |  |  | Zabol | 0/5 |  |  |  |  |  |
|  |  |  |  |  |  |  |  |  | Zehak | 0/6 |  |  |  |  |  |
|  |  |  |  |  |  |  |  |  | Hamun | 0/2 |  |  |  |  |  |
|  |  |  |  |  |  |  |  |  | Hirmand | 0/3 |  |  |  |  |  |
|  |  |  |  |  |  |  |  |  |  |  |  |  |  |  |  |
| 23 |  | *Passer domesticus* (Linnaeus, 1758) | House sparrow | Least concern | گنجشک خانگی | *-* | - | - | Shirvan | 0/3 |  |  |  |  |  |
|  |  |  |  |  |  |  |  |  | Gorgan | 0/1 |  |  |  |  |  |
|  |  |  |  |  |  |  |  |  | Kalaleh | 0/4 |  |  |  |  |  |
|  |  |  |  |  |  |  |  |  | Aqqala | 0/4 |  |  |  |  |  |
|  |  |  |  |  |  |  |  |  | Rasht | 0/2 |  |  |  |  |  |
|  |  |  |  |  |  |  |  |  | Sari | 0/1 |  |  |  |  |  |
|  |  |  |  |  |  |  |  |  | Babol | 0/3 |  |  |  |  |  |
|  |  |  |  |  |  |  |  |  | Birjand | 0/3 |  |  |  |  |  |
|  |  |  |  |  |  |  |  |  | Nehbandan | 0/1 |  |  |  |  |  |
|  |  |  |  |  |  |  |  |  | Quchan | 0/1 |  |  |  |  |  |
|  |  |  |  |  |  |  |  |  |  |  |  |  |  |  |  |
| 30 |  | *Passer montanus* (Linnaeus, 1758) | Eurasian tree sparrow | Least concern | گنجشک درختی | *-* | - | - | Hamun | 0/7 |  |  |  |  |  |
|  |  |  |  |  |  |  |  |  | Zabol | 0/9 |  |  |  |  |  |
|  |  |  |  |  |  |  |  |  | Hirmand | 0/3 |  |  |  |  |  |
|  |  |  |  |  |  |  |  |  | Zehak | 0/5 |  |  |  |  |  |
|  |  |  |  |  |  |  |  |  | Nimruz | 0/6 |  |  |  |  |  |
|  |  |  |  |  |  |  |  |  |  |  |  |  |  |  |  |
| 21 | **Pycnonotidae** | *Pycnonotus leucotis* (Gould, 1836) | White-eared bulbul | Least concern | بلبل خرما | *-* | - | - | Hamun | 0/4 |  |  |  |  |  |
|  |  |  |  |  |  |  |  |  | Zabol | 0/6 |  |  |  |  |  |
|  |  |  |  |  |  |  |  |  | Hirmand | 0/4 |  |  |  |  |  |
|  |  |  |  |  |  |  |  |  | Zehak | 0/5 |  |  |  |  |  |
|  |  |  |  |  |  |  |  |  | Nimruz | 0/2 |  |  |  |  |  |
|  |  |  |  |  |  |  |  |  |  |  |  |  |  |  |  |
| 4 | **Laniidae** | *Lanius phoenicuroides* (Schalow, 1875) | Red-tailed shrike | Least concern | سنگ چشم طورانی | *-* | - | - | Hirmand | 0/1 |  |  |  |  |  |
|  |  |  |  |  |  |  |  |  | Hamun | 0/1 |  |  |  |  |  |
|  |  |  |  |  |  |  |  |  | Zehak | 0/1 |  |  |  |  |  |
|  |  |  |  |  |  |  |  |  | Zabol | 0/1 |  |  |  |  |  |
|  |  |  |  |  |  |  |  |  |  |  |  |  |  |  |  |
| 14 | **Leiothrichidae** | *Turdoides caudata* (Dumont, 1823) | Common babbler | Least concern | دومیل (یا) لیکوی معمولی | *-* | - | - | Hamun | 0/4 |  |  |  |  |  |
|  |  |  |  |  |  |  |  |  | Zabol | 0/3 |  |  |  |  |  |
|  |  |  |  |  |  |  |  |  | Hirmand | 0/2 |  |  |  |  |  |
|  |  |  |  |  |  |  |  |  | Zehak | 0/3 |  |  |  |  |  |
|  |  |  |  |  |  |  |  |  | Nimruz | 0/2 |  |  |  |  |  |
|  |  |  |  |  |  |  |  |  |  |  |  |  |  |  |  |
| 11 | **Motacillidae** | *Motacilla alba* Linnaeus, 1758 | White wagtail | Least concern | دم جنبانک ابلق | *-* | - | - | Gorgan | 0/1 |  |  |  |  |  |
|  |  |  |  |  |  |  |  |  | Quchan | 0/1 |  |  |  |  |  |
|  |  |  |  |  |  |  |  |  | Bojnurd | 0/1 |  |  |  |  |  |
|  |  |  |  |  |  |  |  |  | Neishaboor | 0/1 |  |  |  |  |  |
|  |  |  |  |  |  |  |  |  | Mashhad | 0/1 |  |  |  |  |  |
|  |  |  |  |  |  |  |  |  | Zabol | 0/1 |  |  |  |  |  |
|  |  |  |  |  |  |  |  |  | Qaen | 0/1 |  |  |  |  |  |
|  |  |  |  |  |  |  |  |  | Nehbandan | 0/1 |  |  |  |  |  |
|  |  |  |  |  |  |  |  |  | Zehak | 0/2 |  |  |  |  |  |
|  |  |  |  |  |  |  |  |  | Hamun | 0/1 |  |  |  |  |  |
|  |  |  |  |  |  |  |  |  |  |  |  |  |  |  |  |
| 7 | **Muscicapidae** | *Oenanthe albonigra* (Hume, 1872) | Hume's wheatear | Least concern | چک چک سر سیاه | *-* | - | - | Zehak | 0/2 |  |  |  |  |  |
|  |  |  |  |  |  |  |  |  | Hamun | 0/1 |  |  |  |  |  |
|  |  |  |  |  |  |  |  |  | Zabol | 0/4 |  |  |  |  |  |
|  |  |  |  |  |  |  |  |  |  |  |  |  |  |  |  |
| 1 |  | *Cercotrichas* *galactotes* ([Temminck](https://en.wikipedia.org/wiki/Coenraad_Jacob_Temminck), 1820) | Rufous-tailed scrub robin | Least concern | دم‌چتری دم‌حنایی | - | - | - | Zabol | 0/1 |  |  |  |  |  |
|  |  |  |  |  |  |  |  |  |  |  |  |  |  |  |  |
| 5 | **Scotocercidae** | *Scotocerca inquieta* (Cretzschmar, 1830) | Streaked scrub warbler | Least concern | سسک جنبان | *-* | - | - | Zehak | 0/2 |  |  |  |  |  |
|  |  |  |  |  |  |  |  |  | Hamun | 0/1 |  |  |  |  |  |
|  |  |  |  |  |  |  |  |  | Zabol | 0/2 |  |  |  |  |  |
|  | PELECANIFORMES |  |  |  | پلیکان­سانان |  |  |  |  |  |  |  |  |  |  |
| 6 | **Ardeidae** | *Ardea cinerea* Linnaeus, 1758 | Gray heron | Least concern | حواصیل خاکستری | *-* | - | - | Hamedan | 0/1 |  |  |  |  |  |
|  |  |  |  |  |  |  |  |  | Zehak | 0/5 |  |  |  |  |  |
|  |  |  |  |  |  |  |  |  |  |  |  |  |  |  |  |
| 5 |  | *Ardea alba* Linnaeus, 1758 | Great egret | Least concern | حواصیل سفید (یا) اگرت بزرگ |  |  |  | Zehak | 0/3 |  |  |  |  |  |
|  |  |  |  |  |  |  |  |  | Hamun | 0/2 |  |  |  |  |  |
|  |  |  |  |  |  |  |  |  |  |  |  |  |  |  |  |
| 1 |  | *Botaurus stellaris* Linnaeus, 1758 | Great Bittern (Eurasian bittern) | Least concern | بوتیمار | *-* | - | - | Hamedan | 0/1 |  |  |  |  |  |
|  |  |  |  |  |  |  |  |  |  |  |  |  |  |  |  |
| 2 |  | *Ixobrychus minutus* (Linnaeus, 1766) | Little bittern | Least concern | بوتیمار کوچک | *-* | - | - | Zahedan | 0/2 |  |  |  |  |  |
|  |  |  |  |  |  |  |  |  |  |  |  |  |  |  |  |
|  | **Pelecanidae** |  |  |  | مرغ سقاییان |  |  |  |  |  |  |  |  |  |  |
| 4 |  | *Pelecanus crispus* Bruch, 1832 | Dalmatian pelican | Vulnerable | پلیکان پا خاکستری | *Colpocephalum eucarenum* Burmeister, 1838 | Amblycera | Menoponidae | Zehak | 0/2 | 6 | 5 | 5 | 0 | 16 |
|  |  |  |  |  |  |  |  |  | Zabol | 1/2 |  |  |  |  |  |
|  | PHOENICOPTERIFORMES |  |  |  | بال­آتشی­سانان |  |  |  |  |  |  |  |  |  |  |
| 3 | **Phoenicopteridae** | *Phoenicopterus ruber* (Linnaeus, 1758) | American flamingo | Least concern | فلامینگو بزرگ | *Colpocephalum heterosoma* Piaget, 1880, small specimen, (Clay, 1951) | Amblycera | Menoponidae | Zabol | 1/1 | 1 | 3 | 0 | 0 | 4 |
|  |  |  |  |  |  | *Colpocephalum heterosoma* Piaget, 1880, large specimen |  |  | Zehak | 0/2 | 1 | 0 | 0 | 0 | 1 |
|  | PODICIPEDIFORMES |  |  |  | کشیم­سانان |  |  |  |  |  |  |  |  |  |  |
| 9 | **Podicipedidae** | *Podiceps cristatus* (Linnaeus, 1758) | Great crested grebe | Least concern | کشیم بزرگ | - | - | - | Zehak | 0/6 |  |  |  |  |  |
|  |  |  |  |  |  |  |  |  | Zabol | 0/3 |  |  |  |  |  |
|  | PTEROCLIDIFORMES |  |  |  | کوکورشکلان |  |  |  |  |  |  |  |  |  |  |
| 1 | **Pteroclidae** | *Pterocles orientalis* (Linnaeus, 1758) | Black-bellied Sandgrouse | Least concern | کوکر شکم سیاه (یا) باقرقره | *-* | - | - | Hamedan | 0/1 |  |  |  |  |  |
|  | PICIFORMES |  |  |  | دارکوب­ها |  |  |  |  |  |  |  |  |  |  |
| 1 | **Picidae** | *Dendrocopos syriacus* (Hemprich & Ehrenberg, 1833) | Syrian woodpecker | Least concern | دارکوب سوری (یا) دارکوب باغی | *-* | - | - | Hamedan | 0/1 |  |  |  |  |  |
|  | SULIFORMES |  |  |  | بوبی‌سانان |  |  |  |  |  |  |  |  |  |  |
| 7 | **Phalacrocoracidae** | *Phalacrocorax carbo* (Linnaeus, 1758) | Great cormorant | Least concern | باکلان بزرگ (یا) نرو | - | - | - | Zabol | 0/2 |  |  |  |  |  |
|  |  |  |  |  |  |  |  |  | Zehak | 0/2 |  |  |  |  |  |
|  |  |  |  |  |  |  |  |  | Chabahar | 0/3 |  |  |  |  |  |
|  | STRIGIFORMES |  |  |  | جغد شکلان |  |  |  |  |  |  |  |  |  |  |
| 2 | **Strigidae** | *Asio otus* (Linnaeus, 1758) | Long-eared owl | Least concern | جغد گوش دراز | *Strigiphilus* sp. | Ischnocera | Philopteridae | Hamedan | 1/2 | 0 | 1 | 0 | 0 | 1 |
|  |  |  |  |  |  |  |  |  |  |  |  |  |  |  |  |
| 9 |  | *Athene noctua* (Scopoli, 1769) | Little owl | Least concern | جغد کوچک | *-* | - | - | Hamedan | 0/1 |  |  |  |  |  |
|  |  |  |  |  |  |  |  |  | Kerman | 0/4 |  |  |  |  |  |
|  |  |  |  |  |  |  |  |  | Shahrekord | 0/2 |  |  |  |  |  |
|  |  |  |  |  |  |  |  |  | Khash | 0/2 |  |  |  |  |  |
|  |  |  |  |  |  |  |  |  |  |  |  |  |  |  |  |
| 8 |  | *Bubo bubo* (Linnaeus, 1758) | Eagle owl | Least concern | شاه بوف | *Strigiphilus strigis* (Pontoppidan, 1763) | Ischnocera | Philopteridae | Hamedan | 2/2 | 26 | 29 | 0 | 0 | 55 |
|  |  |  |  |  |  |  |  |  | Zahedan | 0/1 |  |  |  |  |  |
|  |  |  |  |  |  |  |  |  | Zabol | 0/1 |  |  |  |  |  |
|  |  |  |  |  |  |  |  |  | Kerman | 0/4 |  |  |  |  |  |
|  |  |  |  |  |  |  |  |  |  |  |  |  |  |  |  |
| 1 |  | *Otus scops* (Linnaeus, 1758) | European scops owl | Least concern | مرغ حق | *-* | - | - | Hamedan | 0/1 |  |  |  |  |  |
|  |  |  |  |  |  |  |  |  |  |  |  |  |  |  |  |
| 2 |  | *Otus brucei* (Hume, 1873) | Pallid scops owl | Least concern | مرغ حق جنوبی | *-* | - | - | Kerman | 0/2 |  |  |  |  |  |
|  |  |  |  |  |  |  |  |  |  |  |  |  |  |  |  |
| 4 | **Tytonidae** | *Tyto alba* (Scopoli, 1769) | Barn owl | Least concern | جغد انبار | *-* | - | - | Hamedan | 0/1 |  |  |  |  |  |
|  |  |  |  |  |  |  |  |  | Kerman | 0/2 |  |  |  |  |  |
|  |  |  |  |  |  |  |  |  | Zahedan | 0/1 |  |  |  |  |  |
|  |  |  |  |  |  |  |  |  |  |  |  |  |  |  |  |
| **Total 612** |  |  |  |  |  |  |  |  |  | **58** | **157** | **157** | **35** | **2** | **352** |

^a^ according to International Union for Conservation of Nature (IUCN) Red List of Threatened Species ([www.iucnredlist.org](http://www.iucnredlist.org)).
