# Supplementary material for: Chewing lice of wild birds in Iran: new data and a checklist of avian louse species reported in Iran
Source: Front Vet Sci. 2024 Feb 2;10:1324619. doi: 10.3389/fvets.2023.1324619 (PMC10869535; doi:10.3389/fvets.2023.1324619)
Supplement: Supplementary file 2 [file Table_2.docx]

**Supplementary Table 2.** Louse species reported from Iran according to their avian hosts until December 2023. Names of orders are capitalized, and names of families are showed in bold.

| **Avian host scientific name** | **Avian host vernacular name** | **Global conservation status^a^** | **نام فارسی** | **Lice species** | **Reference** |
| --- | --- | --- | --- | --- | --- |
| **ACCIPITRIFORMES** |  |  | بازسانان |  |  |
| **Accipitridae** |  |  | بازان |  |  |
| *Aquila chrysaetos* (Linnaeus, 1758) | Golden eagle | Least Concern | عقاب طلایی | *Craspedorrhynchus aquilinus* (Denny, 1842) | (Rak et al*.*, [1975](#Rak1975); Azizi et al*.*, [2013](#azizi2013)), this study |
|  |  |  |  | *Laemobothrion maximum* (Scopoli, 1763) |  |
|  |  |  |  | *Laemobothrion* sp. |  |
|  |  |  |  |  |  |
| *Aquila fasciata* (Vieillot, 1822) | Bonelli’s eagle | Least Concern | عقاب دو برادر | *Laemobothrion maximum* (Scopoli, 1763) | (Alborzi & Naddaf, [2008](#alborzi2008)). Reported the bird as *Hieraaetus fasciatus* |
|  |  |  |  |  |  |
| *Aquila heliaca* Savigny, 1809 | Asian imperial eagle | Vulnerable | عقاب شاهی | *Laemobothrion maximum* (Scopoli, 1763) | This study |
|  |  |  |  |  |  |
| *Aquila nipalensis* Hodgson, 1833 | Steppe eagle | Endangered | عقاب صحرایی | *Colpocephalum impressum* Rudow, 1866 | (Ahoo et al*.*, [2018](#Ahoo2018)), this study |
|  |  |  |  | *Falcolipeurus suturalis* (Rudow, 1869) |  |
|  |  |  |  | *Laemobothrion maximum* (Scopoli, 1763) |  |
|  |  |  |  | *Craspedorrhynchus* sp. |  |
|  |  |  |  |  |  |
| *Aquila rapax* (Temminck, 1828) | Tawny eagle | Least Concern | عقاب خاکی (دشتی) | *Laemobothrion vulturis* (Fabricius, 1775) | This study |
|  |  |  |  | *Colpocephalum impressum* Rudow, 1866 |  |
|  |  |  |  | *Nosopon chanabense* (Ansari, 1951) |  |
|  |  |  |  |  |  |
| *Buteo buteo* (Linnaeus, 1758) | Buzzard | Least Concern | سارگپه | *Degeeriella fulva* (Giebel, 1874) | This study |
|  |  |  |  | *Degeeriella fusca* (Denny, 1842) |  |
|  |  |  |  | *Cuclotogaster heterographus* (Nitzsch, 1866) |  |
|  |  |  |  | *Craspedorrhynchus platystomus* (Burmeister, 1838) |  |
|  |  |  |  | *Colpocephalum nanum* Piaget, 1890 |  |
|  |  |  |  | *Colpocephalum turbinatum* Denny, 1842 |  |
|  |  |  |  | *Laemobothrion maximum* (Scopoli, 1763) |  |
|  |  |  |  |  |  |
| *Buteo rufinus* (Cretzschmar, 1829) | Long-legged buzzard | Least Concern | سارگپه پا بلند | *Trinoton* sp.^×^ | (Ghaemi et al*.*, [2010](#ghasemi2010)), this study |
|  |  |  |  | *Laemobothrion maximum (*Scopoli, 1763*)* |  |
|  |  |  |  |  |  |
| *Circus aeruginosus* (Linnaeus, 1758) | Eurasian marsh-harrier | Least Concern | سنقر تالابی | *Nosopon lucidum* (Rudow, 1869) | This study |
|  |  |  |  |  |  |
| *Gyps fulvus* (Hablizl, 1783) | Eurasian griffon vulture | Least Concern | دال | *Trinoton* sp.^×^ | (Ghaemi & Roshanian, [2009](#ghasemi2009)), this study |
|  |  |  |  | *Laemobothrion vulturis* (Fabricius, 1775) |  |
|  |  |  |  | *Colpocephalum gypsi* (Eichler & Zlotorzycka, 1971) |  |
|  |  |  |  | *Colpocephalum* spp. |  |
|  |  |  |  | *Falcolipeurus quadripustulatus* (Burmeister, 1838) |  |
|  |  |  |  | *Aegypoecus trigonoceps* (Giebel, 1874) |  |
|  |  |  |  |  |  |
| *Neophron percnopterus* (Linnaeus, 1758) | Egyptian vulture | Endangered | کرکس کوچک یا کرکس مصری | *Laemobothrion vulturis* (Fabricius, 1775) | (Ardalan, [1972](#ardalan1972)) |
|  |  |  |  |  |  |
| **ANSERIFORMES** |  |  | غازسانان |  |  |
| **Anatidae** |  |  | مرغابیان |  |  |
| *Anas clypeata* Linnaeus, 1758 | Northern shoveler | Least Concern | اردک نوک پهن | *Anaticola crassicornis* (Scopoli, 1763) | (Rak et al*.*, [1975](#Rak1975)), this study |
|  |  |  |  | *Pectinopygus* spp. |  |
|  |  |  |  |  |  |
| *Anas crecca* Linnaeus, 1758 | Common teal | Least Concern | خوتکای معمولی | *Trinoton querquedulae* (Linnaeus, 1758) | This study |
|  |  |  |  | *Anaticola crassicornis* (Scopoli, 1763) |  |
|  |  |  |  |  |  |
| *Anas platyrhynchos* Linnaeus, 1758 | Mallard | Least Concern | مرغابی سَرسَبز، اردک سرسبز، سرسبز یا کلّه‌سبز | *Lipeurus squalidus* Piaget, 1880^×^ | (Rafyi et al*.*, [1968](#Rafyi1968); Shemshadi et al*.*, [2017](#Shemshadi2017)), this study |
|  |  |  |  | *Menacanthus stramineus* (Nitzsch, 1818)^×^ |  |
|  |  |  |  | *Trinoton anserinum* (Fabricius, 1805)^×^ |  |
|  |  |  |  | *Anatolica crassicornis (Scopoli,* 1763*)* |  |
|  |  |  |  |  |  |
| *Anser anser* (Linnaeus, 1758) | Greylag goose | Least Concern | غاز خاکستری | *Anaticola anseris* (Linnaeus, 1758) | (Rafyi et al*.*, [1968](#Rafyi1968); Ardalan, [1972](#ardalan1972); Hosseini et al*.*, [2001](#hosseini2001)) |
|  |  |  |  | *Trinoton anserinum* (Fabricius, 1805) |  |
|  |  |  |  | *Cuclotogaster heterographus* (Nitzsch, 1866) ^×^. Also reported as *Liperus heterographus*^×^ |  |
|  |  |  |  | *Lipeurus caponis* (Linnaeus, 1758)^×^ |  |
|  |  |  |  | *Menopon gallinae* (Linnaeus, 1758)^×^ |  |
|  |  |  |  |  |  |
| *Mareca penelope* (Linnaeus, 1758) | Eurasian wigeon | Least Concern | گیلار اوراسیایی | *Laemobothrion* spp. | This study |
|  |  |  |  |  |  |
| *Spatula querquedula* (Linnaeus, 1758) | Garganey | Least Concern | خوتکای ابروسفید | *Trinoton querquedualea* (Linnaeus, 1758) | This study |
|  |  |  |  |  |  |
| **BUCEROTIFORMES** |  |  | نوک شاخ­سانان |  |  |
| **Upupidae** |  |  | هدهدیان |  |  |
| *Upupa epops* Linnaeus, 1758 | Hoopoe | Least Concern | هدهد یا شانه به سر | *Upupicola upupae* (Schrank, 1803) | (Rak et al*.*, [1975](#Rak1975)) |
|  |  |  |  |  |  |
| **CHARADRIIFORMES** |  |  | سلیم­سانان |  |  |
| **Laridae** |  |  | کاکاییان |  |  |
| *Chroicocephalus ridibundus* (Linnaeus, 1766) | Black-headed gull | Least Concern | کاکایی سرسیاه کوچک | *Austromenopon transversum* (Denny, 1842) | (Dik & Halajian, [2013](#dik2013)). Reported the bird as *Larus ridibundus* |
|  |  |  |  |  |  |
| *Sterna hirundo* (Linnaeus, 1758) | Tern | Least Concern | پرستودریایی معمولی | *Quadraceps legatus* Timmermann, 1952 | (Ardalan, [1975](#ardalan1975)), this study |
|  |  |  |  | *Saemundssonia meridiana* Timmermann, 1950 | This study |
|  |  |  |  |  |  |
| **Recurvirostridae** |  |  | نوک خنجری­ها |  |  |
| *Himantopus* *himantopus* (Linnaeus, 1758) | Black-winged stilt | Least Concern | چوب پای بال سیاه | *Actornithophilus* *uniseriatus* (Piaget, 1880)  *Quadraceps* spp. | This study |
|  |  |  |  |  |  |
| **Scolopacidae** |  |  | آبچلیکان |  |  |
| *Calidris pugnax* (Linnaeus, 1758) | Ruff | Least Concern | تلیله شکیل | *Lunaceps* *holophaeus* Burmeister, 1838 | This study |
|  |  |  |  | *Actornithophilus* *cornutus* (Giebel, 1866) |  |
|  |  |  |  |  |  |
| *Numenius arquata* (Linnaeus, 1758) | Curlew | Near Threatened | گیلانشاه بزرگ | *Cummingsiella ovalis* (Scopoli, 1763) | (Ardalan, [1971](#ardalan1971), [1972](#ardalan1972), [1975](#ardalan1975)) |
|  |  |  |  | *Quadraceps obtusus* (Kellogg & Kuwana, 1902) |  |
|  |  |  |  | *Saemundssonia scolopacis phaeopodis* subsp*. humeralis* (Denny, 1842) |  |
|  |  |  |  |  |  |
| *Scolopax rusticola* (Linnaeus, 1758) | Eurasian woodcock | Least Concern | ابیای اوراسیایی | *Lipeurus* sp. | (Youssefi et al*.*, [2018](#Youssefi2018)) |
|  |  |  |  | *Philopterus* sp*.* |  |
|  |  |  |  |  |  |
| *Tringa stagnatilis* (Bechstein, 1803) | Marsh sandpiper | Least Concern | آبچلیک تالابی | *Quadraceps obscurus* (Burm, 1838) | This study |
|  |  |  |  |  |  |
| **COLUMBIFORMES** |  |  | کبوترسانان |  |  |
| **Columbidae** |  |  | کبوتران |  |  |
| *Columba livia* subsp. *domestica* Gmelin, 1789 | Domestic pigeon | Least Concern | کبوتر رمیده یا کبوتر شهری (خیابانی) | *Campanulotes compar* (Burmeister, 1838). Also reported as *Goniocotes bidentatus* | (Rafyi et al*.*, [1968](#Rafyi1968); Radfar et al*.*, [2012](#Radfar2012); Borji et al*.*, [2013](#borji2013); Rezaei et al*.*, [2016](#Rezaei2016)) |
|  |  |  |  | *Columbicola columbae* (Linnaeus, 1758) |  |
|  |  |  |  | *Columbicola tschulyschman* (Eichler, 1942) |  |
|  |  |  |  | *Lipeurus caponis* (Linnaeus, 1758)^×^ |  |
|  |  |  |  | *Menopon gallinae* (Linnaeus, 1758)^×^ |  |
|  |  |  |  | *Menacanthus stramineus* (Nitzsch, 1818)^×^. Also reported as *Menopon stramineum*^×^ |  |
|  |  |  |  |  |  |
| *Columba livia* subsp. *livia* Gmelin, 1789 | Rock dove | Least Concern | کبوتر چاهی | *Campanulotes compar* (Burmeister, 1838) | (Dik & Halajian, [2013](#dik2013); Chaechi-Nosrati et al*.*, [2018](#ChaechiNosrati2018)) |
|  |  |  |  | *Colpocephalum turbinatum* Denny, 1842 |  |
|  |  |  |  | *Columbicola columbae* (Linnaeus, 1758) |  |
|  |  |  |  | *Hohorstiella lata* (Piaget, 1880) |  |
|  |  |  |  | *Menacanthus stramineus* (Nitzsch, 1818)^×^ |  |
|  |  |  |  | *Menopon gallinae* (Linnaeus, 1758)^×^ |  |
|  |  |  |  | *Goniodes* sp. |  |
|  |  |  |  |  |  |
| *Streptopelia senegalensis* (Linnaeus, 1766) | Laughing dove | Least Concern | قمری خانگی | *Columbicola columbae* (Linnaeus, 1758) | (Mahmoudian, [2015](#Mahmoudian2015)) |
|  |  |  |  |  |  |
| *Streptopelia turtur* (Linnaeus, 1758) | European turtle dove | Vulnerable | قُمری معمولی | *Colpocephalum pectinatum* (Osborn, 1902) | (Rak et al*.*, [1975](#Rak1975)) |
|  |  |  |  | *Strigiphilus* sp.^×^ |  |
|  |  |  |  |  |  |
| **CORACIIFORMES** |  |  | سبزقباسانان |  |  |
| **Alcedinidae** |  |  | ماهی‌خورک‌ها |  |  |
| *Alcedo atthis* (Linnaeus, 1758) | Common kingfisher | Least Concern | ماهی‌خورک معمولی | *Alcedoecus annulatus* Ansari, 1955 | (Rak et al*.*, [1975](#Rak1975)) |
|  |  |  |  |  |  |
| **Meropidae** |  |  | زنبورخواران |  |  |
| *Merops apiaster* Linnaeus, 1758 | Bee-eater | Least Concern | زنبورخوار معمولی | *Meromenopon meropis* Clay & Meinertzhagen, 1941 | (Nazarbeigy et al*.*, [2019](#Nazarbeigi2019)) |
|  |  |  |  | *Meropoecus meropis* (Denny, 1842) |  |
|  |  |  |  | *Meropsilla apiastri* (Denny, 1842). Reported as *Brueelia* *apiastri* |  |
|  |  |  |  |  |  |
| *Merops persicus* Pallas, 1773 | Blue-cheeked bee-eater | Least Concern | زنبورخوار گلوخرمایی | *Meromenopon meropis* Clay & Meinertzhagen, 1941 | (Nazarbeigy et al*.*, [2019](#Nazarbeigi2019)) |
|  |  |  |  | *Meropoecus meropis* (Denny, 1842) |  |
|  |  |  |  | *Meropsiella erythropteri* (Piaget, 1885). Reported as *Brueelia erythropteri* |  |
|  |  |  |  |  |  |
| **CUCULIFORMES** |  |  | کوکوسانان |  |  |
| **Cuculidae** |  |  | کوکویان |  |  |
| *Cuculus canorus* Linnaeus, 1758 | Common cuckoo | Least Concern | کوکوی معمولی، کوکوی اروپایی (یا) فاخته | *Cuculoecus latifrons* (Denny, 1842). Reported as *Philopterus latifron* | (Ardalan, [1971](#ardalan1971)) |
|  |  |  |  |  |  |
| **FALCONIFORMES** |  |  | شاهین­سانان |  |  |
| **Falconidae** |  |  | شاهینان |  |  |
| *Falco cherrug* J.E.Gray, 1834 | Saker falcon | Endangered | بالابان | *Colpocephalum* sp. | (Rak et al*.*, [1975](#ardalan1975)) |
|  |  |  |  |  |  |
| *Falco tinnunculus* Linnaeus, 1758 | Common kestrel | Least Concern | دلیجه معمولی | *Laemobothrion maximum* (Scopoli, 1763)^×^ | (Rak et al*.*, [1975](#Rak1975)), this study |
|  |  |  |  |  |  |
| **GALLIFORMES** |  |  | ماکیان‌سانان |  |  |
| **Phasianidae** |  |  | قرقاولان |  |  |
| *Coturnix coturnix* (Linnaeus, 1758) | Common quail | Least Concern | بلدرچین معمولی | *Amyrsidea fulvomaculata* (Denny, 1842) | (Rak et al*.*, [1975](#Rak1975)) |
|  |  |  |  |  |  |
| *Gallus gallus domesticus* (Linnaeus, 1758) | Domestic chicken | Least Concern | مُرغ و خروس خانگی | *Cuclotogaster heterographus* (Nitzsch, 1866). Also reported as *Lipeurus heterographus* | (Oormazdi, [1958](#Oormazdi1958); Maghami, [1968](#Maghami1968); Rafyi et al*.*, [1968](#Rafyi1968); Vazirianzadeh et al*.*, [2007](#Vazirianzadeh2007); Eslami et al*.*, [2009](#eslami2009); Hashemzadeh-Farhang et al., [2009](#hashemzadeh2009); Mamashly et al., [2010](#Mamashly2010); Nazarbeigy et al*.*, [2013](#Nazarbeigy2013); Ebrahimi et al., [2016](#ebrahimi2016); Rezaei et al., [2016](#Rezaei2016); Zakian et al*.*, [2016](#Zakian2016); Hossienzadeh Marzenaki, [2017](#hosseinzadeh2017), Shamsi et al*.*, [2020](#Shamsi2020))^b^ |
|  |  |  |  | *Goniodes dissimilis* Denny, 1842 |  |
|  |  |  |  | *Goniocotes gallinae* (de Geer, 1778) |  |
|  |  |  |  | *Goniodes gigas* (Taschenberg, 1879). Also reported as *Goniocotes gigas* |  |
|  |  |  |  | *Lipeurus caponis* (Linnaeus, 1758) |  |
|  |  |  |  | *Menacanthus pallidulus* (Neumann, 1912). Also reported as *Menopon pallidulum* |  |
|  |  |  |  | *Menacanthus stramineus* (Nitzsch, 1818). Also reported as *Menopon stramineum* |  |
|  |  |  |  | *Menopon gallinae* (Linnaeus, 1758) |  |
|  |  |  |  | *Goniodes* sp. |  |
|  |  |  |  | *Lipeurus* sp. |  |
|  |  |  |  | *Menopon* sp. |  |
|  |  |  |  |  |  |
| *Meleagris gallopavo* Linnaeus, 1758 | Common turkey | Least Concern | بوقلمون معمولی | *Chelopistes meleagridis* (Linnaeus, 1758) | (Rafyi et al*.*, [1968](#Rafyi1968); Rassouli et al*.*, [2016](#Rassouli2016); Rezaei et al., [2016](#Rezaei2016)) |
|  |  |  |  | *Goniocotes gallinae* (de Geer, 1778) |  |
|  |  |  |  | *Goniodes gigas* (Taschenberg, 1879) |  |
|  |  |  |  | *Menacanthus stramineus* (Nitzsch, 1818) |  |
|  |  |  |  | *Menopon gallinae* (Linnaeus, 1758) |  |
|  |  |  |  |  |  |
| *Phasianus colchicus* Linnaeus, 1758 | Common pheasant | Least Concern | قرقاول معمولی (یا) تورنگ | *Amyrsidea perdicis* (Denny, 1842). Reported as *Amyrsidea hexapilosus* | (Rak et al., [1975](#Rak1975)) |
|  |  |  |  |  |  |
| *Pavo cristatus* Linnaeus, 1758 | Common peafowl | Least Concern | طاووس معمولی | *Goniodes pavonis* (Linnaeus, 1758) | (Ganjali et al*.*, [2015](#ganjali2015)) |
|  |  |  |  |  |  |
| *Perdix perdix* (Linnaeus, 1758) | Grey partridge | Least Concern | کبک خاکستری | *Lipeurus* sp. | (Sadaghian & Nouri, [2014](#Sadaghian2014)) |
|  |  |  |  | *Menacanthus* sp. |  |
|  |  |  |  | *Menopon* sp. |  |
|  |  |  |  |  |  |
| **GRUIFORMES** |  |  | درناشکلان |  |  |
| **Rallidae** |  |  | یَلوگان |  |  |
| *Rallus aquaticus* Linnaeus, 1758 | Water rail | Least Concern | یلوه آبی | *Rallicola cuspidatus* (Scopoli, 1763) | This study |
|  |  |  |  |  |  |
| *Fulica atra* Linnaeus, 1758 | Coot | Least Concern | چنگر اوراسیایی | *Laemobothrion* *atrum* (Nitzsch, 1818) | This study |
|  |  |  |  |  |  |
| **PASSERIFORMES** |  |  | گنجشک­سانان |  |  |
| **Acrocephalidae** |  |  | سسک‌های نیزار |  |  |
| *Acrocephalus stentoreus* (Hemprich & Ehrenberg, 1833) | Clamorous reed warbler | Least Concern | سسک نیزار پر صدا (تالابی) | *Brueelia* sp. | (Moodi et al*.*, [2013](#Moodi2013)) |
|  |  |  |  |  |  |
| **Alaudidae** |  |  | چکاوک­ها |  |  |
| *Calandrella rufescens* (Vieillot, 1819) | Lesser short-toed lark | Least Concern | چکاوک کوچک (سینه‌ خط‌ دار) | *Menacanthus* sp*.* | (Moodi et al., [2013](#Moodi2013)) |
|  |  |  |  |  |  |
| *Galerida cristata* (Linnaeus, 1758) | Crested lark | Least Concern | چکاوک کاکلی | *Brueelia* sp. | (Moodi et al*.*, [2013](#Moodi2013)) |
|  |  |  |  | *Ricinus* sp. |  |
|  |  |  |  |  |  |
| **Corvidae** |  |  | کَلاغان |  |  |
| *Corvus corax* Linnaeus, 1758 | Common raven | Least Concern | غراب معمولی | *Myrsidea anaspila* (Nitzsch, 1866) | (Ardalan, [1971](#ardalan1971), [1972](#ardalan1972)) |
|  |  |  |  | *Philopterus corvi* (Linnaeus, 1758) |  |
|  |  |  |  | *Cuclotogaster heterographus* (Nitzsch, 1866)^×^ |  |
|  |  |  |  |  |  |
| *Corvus corone* Linnaeus, 1758 | Carrion crow | Least Concern | کلاغ لاشه‌خوار | *Philopterus ocellatus* (Scopoli, 1763) | (Ardalan, [1971](#ardalan1971); Moodi et al*.*, [2013](#Moodi2013); Imanibaran, [2014](#Imanibaran2014)) |
|  |  |  |  | *Brueelia* sp. |  |
|  |  |  |  | *Cuculoecus latifrons* (Denny, 1842). Also reported as *Philopterus latifron*^×^ |  |
|  |  |  |  |  |  |
| *Pica pica* (Linnaeus, 1758) | Black-billed magpie | Least Concern | زاغی اوراسیایی | *Philopterus picae* (Denny, 1842) | (Rak et al*.*, [1975](#Rak1975)) |
|  |  |  |  |  |  |
| **Emberizidae** |  |  | زردپرگان |  |  |
| *Emberiza bruniceps* J.F.Brandt, 1841 | Red-headed bunting | Least Concern | زردپره سرسرخ | *Sturnidoecus rostratus* (Mey, 1982) | (Moodi et al*.*, [2013](#Moodi2013)) |
|  |  |  |  | *Menacanthus* sp*.* |  |
|  |  |  |  |  |  |
| *Emberiza calandra* Linnaeus, 1758 | Corn bunting | Least Concern | زردپره مزرعه | *Sturnidoecus rostratus* (Mey, 1982) | (Moodi et al., [2013](#Moodi2013)) |
|  |  |  |  | *Brueelia* sp. |  |
|  |  |  |  |  |  |
| **Fringillidae** |  |  | سهرگان |  |  |
| *Chloris chloris* (Linnaeus, 1758) | European greenfinch | Least Concern | سهره سبز | *Myrsidea* sp*.* | (Moodi et al*.*, [2013](#Moodi2013)) |
|  |  |  |  |  |  |
| *Rhodospiza obsoleta* (M.H.K.Lichtenstein, 1823) | Desert finch | Least Concern | سهره خاکی | *Brueelia gobiensis* Mey, 1982 | (Moodi et al., [2013](#Moodi2013)). Reported the bird as *Carduelis* *obsoleta* |
|  |  |  |  | *Philopterus* sp*.* |  |
|  |  |  |  |  |  |
| *Fringilla coelebs* Linnaeus, 1758 | Chaffinch | Least Concern | سهره جنگلی | *Philopterus fringillae* (Scopoli, 1772) | (Moodi et al*.*, [2013](#Moodi2013)) |
|  |  |  |  | *Brueelia* sp. |  |
|  |  |  |  |  |  |
| **Muscicapidae** |  |  | مگس‌گیران |  |  |
| *Saxicola torquatus* (Linnaeus, 1766) | African stonechat | Least Concern | چک افریقایی | *Brueelia* sp. | (Moodi et al*.*, [2013](#Moodi2013)) |
|  |  |  |  |  |  |
| *Luscinia megarhynchos* C.L.Brehm, 1831 | Common nightingale | Least Concern | بلبل هزاردستان | *Brueelia* sp. | (Moodi et al., [2013](#Moodi2013)) |
|  |  |  |  |  |  |
| *Oenanthe lugens* (Lichtenstein, 1823) | Mourning wheatear | Least Concern | چکچک ابلق جنوبی | *Philopterus* sp*.* | (Moodi et al., [2013](#Moodi2013)) |
|  |  |  |  |  |  |
| **Paridae** |  |  | چرخ‌ریسکان |  |  |
| *Parus major* Linnaeus, 1758 | Great tit | Least Concern | چرخ‌ریسک بزرگ | *Philopterus pallescens* (Denny, 1842) | (Moodi et al., [2013](#Moodi2013)) |
|  |  |  |  |  |  |
| **Passeridae** |  |  | گنجشک­ها |  |  |
| *Gymnoris xanthocollis* (Burton, 1838) | Yellow-throated sparrow | Least Concern | گنجشک گلوزرد | *Philopterus fringillae* (Scopoli, 1772) | (Moodi et al., [2013](#Moodi2013)). Reported the bird as *Petronia xanthocollis* |
|  |  |  |  |  |  |
| *Passer domesticus* (Linnaeus, 1758) | House sparrow | Least Concern | گنجشک خانگی | *Brueelia cyclothorax* (Burmeister, 1838). Reported as *Brueelia subtilis* (Nitzsch, 1874) | (Moodi et al*.*, [2013](#Moodi2013)) |
|  |  |  |  | *Philopterus fringillae* (Scopoli, 1772) |  |
|  |  |  |  | *Sturnidoecus refractariolus* (Zlotorzycka, 1964) |  |
|  |  |  |  |  |  |
| *Passer montanus* (Linnaeus, 1758) | Eurasian sparrow | Least Concern | گنجشک درختی | *Brueelia cyclothorax* (Burmeister, 1838). Reported as *Brueelia subtilis* (Nitzsch, 1874) | (Moodi et al*.*, [2013](#Moodi2013)) |
|  |  |  |  | *Philopterus montani* (Zlotorzycka, 1964) |  |
|  |  |  |  | *Sturnidoecus ruficeps* (Nitzsch, 1866) |  |
|  |  |  |  | *Campanulotes compar* (Burmeister, 1838)^×^ |  |
|  |  |  |  |  |  |
| *Petronia petronia* (Linnaeus, 1766) | Rock petronia | Least Concern | گنجشک کوهی | *Sturnidoecus refractariolus* (Zlotorzycka, 1964) | (Moodi et al*.*, [2013](#Moodi2013)) |
|  |  |  |  | *Brueelia* sp. |  |
|  |  |  |  | *Philopterus* sp. |  |
|  |  |  |  |  |  |
| **Phylloscopidae** |  |  | سسکان برگی |  |  |
| *Phylloscopus collybita* (Vieillot, 1817) | Chiffchaff | Least Concern | سسک چیف‌چاف | *Brueelia* sp. | (Moodi et al*.*, [2013](#Moodi2013)) |
|  |  |  |  | *Menacanthus* sp*.* |  |
|  |  |  |  | *Philopterus* sp*.* |  |
|  |  |  |  | *Sturnidoecus* sp*.* |  |
|  |  |  |  |  |  |
| *Phylloscopus nitidus* Blyth, 1843 | Green warbler | Least Concern | سسک سبز بیدی | *Brueelia* sp. | (Moodi et al*.*, [2013](#Moodi2013)) |
|  |  |  |  | *Menacanthus* sp*.* |  |
|  |  |  |  |  |  |
| **Sturnidae** |  |  | ساران |  |  |
| *Acridotheres tristis* (Linnaeus, 1766) | Common myna | Least Concern | مینای معمولی | *Brueelia chayanh* Ansari, 1955 | (Moodi et al*.*, [2013](#Moodi2013); Ahoo et al*.*, [2018](#Ahoo2018)) |
|  |  |  |  | *Myrsidea invadens* (Kellogg & Chapman, 1902) |  |
|  |  |  |  |  |  |
| *Sturnus vulgaris* Linnaeus, 1758 | Common starling | Least Concern | سار معمولی | *Brueelia nebulosa* (Burmeister, 1838) | (Moodi et al*.*, [2013](#Moodi2013)) |
|  |  |  |  |  |  |
| **Sylviidae** |  |  | سسکان |  |  |
| *Sylvia communis* Latham, 1787 | Common whitethroat | Least Concern | سسک گلوسفید معمولی | *Sturnidoecus* sp*.* | (Moodi et al*.*, [2013](#Moodi2013)) |
|  |  |  |  |  |  |
| **Turdidae** |  |  | توکایان |  |  |
| *Turdus ruficollis* Pallas, 1776 | Black-throated Thrush | Least Concern | توکای گلوسرخ | *Philopterus* sp*.* | (Rak et al*.*, [1975](#Rak1975)) |
|  |  |  |  |  |  |
| *Turdus merula* Linnaeus, 1758 | Blackbird | Least Concern | توکای سیاه معمولی | *Ricinus* sp. | (Moodi et al*.*, [2013](#Moodi2013)) |
|  |  |  |  |  |  |
| **PELECANIFORMES** |  |  | پلیکان‌سانان |  |  |
| **Ardeidae** |  |  | حواصیل­ها |  |  |
| *Ardea purpurea* Linnaeus, 1766 | Purple heron | Least Concern | حواصیل ارغوانی | *Menacanthus* sp.^×^ | (Dik & Halajian, [2013](#dik2013)) |
|  |  |  |  |  |  |
| *Egretta garzetta* (Linnaeus, 1766) | Little egret | Least Concern | قار کوچک (یا) اگرت کوچک | *Ardeicola* sp. Probably *Ardeicola expallidus* Blagoveshtchensky, 1940 | (Dik & Halajian, [2013](#dik2013)) |
|  |  |  |  | *Ciconiphilus decimfasciatus* (Boisduval & Lacordaire, 1835) |  |
|  |  |  |  |  |  |
| **Pelecanidae** |  |  | مرغ سقاییان |  |  |
| *Pelecanus onocrotalus* (Linnaeus, 1758) | Great white pelican | Least Concern | پلیکان سفید بزرگ | *Piagetiella titan* (Piaget, 1880) | (Tavassoli et al*.*, [2011](#Tavassoli2011)) |
|  |  |  |  |  |  |
| *Pelecanus crispus* (Bruch, 1832) | Dalmatian pelican | Vulnerable | پلیکان خاکستری | *Colpocephalum eucarenum* Burmeister, 1838 | This study |
|  |  |  |  |  |  |
| **PHOENICOPTERIFORMES** |  |  | بال­آتشی­سانان |  |  |
| **Phoenicopteridae** |  |  | بال‌آتشیان |  |  |
| *Phoenicopterus ruber* Linnaeus, 1758 | American flamingo | Least Concern | فلامینگوی بزرگ | *Colpocephalum heterosoma* Piaget, 1880 | This study |
|  |  |  |  |  |  |
| **PODICIPEDIFORMES** |  |  | کشیم سانان |  |  |
| **Podicipedidae** |  |  | کشیمان |  |  |
| *Podiceps cristatus* (Linnaeus, 1758) | Great crested grebe | Least Concern | کشیم کاکلی بزرگ | *Aquanirmus podicipis* (Denny, 1842) | (Dik & Halajian, [2013](#dik2013) ) |
|  |  |  |  | *Pseudomenopon dolium* (Rudow, 1869) |  |
|  |  |  |  |  |  |
| **STRIGIFORMES** |  |  | جغدسانان |  |  |
| **Strigidae** |  |  | جغدان راستین |  |  |
| *Asio otus* (Linnaeus, 1758) | Long-eared owl | Least Concern | جغد گوش‌دراز | *Strigiphilus* sp. | This study |
|  |  |  |  |  |  |
| *Athene noctua* (Scopoli, 1769) | Little owl | Least Concern | جغد کوچک | *Colpocephalum pectinatum* Osborn, 1902 | (Rak et al*.*, [1975](#Rak1975)) |
|  |  |  |  | *Philopterus ocellatus* (Scopoli, 1763)^×^ |  |
|  |  |  |  |  |  |
| *Bubo bubo* (Linnaeus, 1758) | Eagle owl | Least Concern | شاه‌ بوف | *Strigiphilus strigis* (Pontoppidan, 1763) | This study |
|  |  |  |  |  |  |
| **SULIFORMES** |  |  | بوبی­سانان |  |  |
| **Phalacrocoracidae** |  |  | باکلانان |  |  |
| *Phalacrocorax carbo* (Linnaeus, 1758) | Common cormorant | Least Concern | باکلان بزرگ | *Pectinopygus gyricornis* (Denny, 1842) | (Dik & Halajian, [2013](#dik2013)) |

^a^ according to International Union for Conservation of Nature (IUCN) Red List of Threatened Species ([www.iucnredlist.org](http://www.iucnredlist.org)).

^×^ The louse species is not normally found on this bird. Its report is probably due to contamination or misidentification.
